# The Difference of Physiological and Proteomic Changes in Maize Leaves Adaptation to Drought, Heat, and Combined Both Stresses

**DOI:** 10.3389/fpls.2016.01471

**Published:** 2016-10-26

**Authors:** Feiyun Zhao, Dayong Zhang, Yulong Zhao, Wei Wang, Hao Yang, Fuju Tai, Chaohai Li, Xiuli Hu

**Affiliations:** ^1^State Key Laboratory of Wheat and Maize Crop Science, Collaborative Innovation Center of Henan Grain Crops, College of Life Science, Henan Agricultural UniversityZhengzhou, China; ^2^Provincial Key Laboratory of Agrobiology, Institute of Biotechnology, Jiangsu Academy of Agricultural SciencesNanjing, China

**Keywords:** proteomics, iTRAQ labeling, combined drought and heat stress, maize, physiological characterization

## Abstract

At the eight-leaf stage, maize is highly sensitive to stresses such as drought, heat, and their combination, which greatly affect its yield. At present, few studies have analyzed maize response to combined drought and heat stress at the eight-leaf stage. In this study, we measured certain physical parameters of maize at the eight-leaf stage when it was exposed to drought, heat, and their combination. The results showed an increase in the content of H_2_O_2_ and malondialdehyde (MDA), and in the enzyme activities of superoxide dismutase (SOD), ascorbate peroxidase (APX), and glutathione reductase (GR), but a decrease in the quantum efficiency of photosystem II (ΦPSII). The most obvious increase or decrease in physical parameters was found under the combined stress condition. Moreover, to identify proteins differentially regulated by the three stress conditions at the eight-leaf stage, total proteins from the maize leaves were identified and quantified using multiplex iTRAQ-based quantitative proteomic and LC-MS/MS methods. In summary, the expression levels of 135, 65, and 201 proteins were significantly changed under the heat, drought and combined stress conditions, respectively. Of the 135, 65, and 201 differentially expressed proteins, 61, 28, and 16 responded exclusively to drought stress, heat stress, and combined stress, respectively. Bioinformatics analysis implied that chaperone proteins and proteases play important roles in the adaptive response of maize to heat stress and combined stress, and that the leaf senescence promoted by ethylene-responsive protein and ripening-related protein may play active roles in maize tolerance to combined drought and heat stress. The signaling pathways related to differentially expressed proteins were obviously different under all three stress conditions. Thus, the functional characterization of these differentially expressed proteins will be helpful for discovering new targets to enhance maize tolerance to stress.

## Introduction

Under field conditions, crops are often subjected to a combination of several stresses, which have an adverse effect or may even prove lethal. Recently, researchers have begun to pay more attention to the potential molecular mechanisms involved in crop endurance to combined stress (Rampino et al., 2012; Liu et al., 2015; Obata et al., 2015). The evidence shows that crops exhibit unique physiological and molecular responses to combined stress, which cannot be directly inferred from plant responses to single stresses. Moreover, the simultaneous occurrence of several stresses brings about a complexity of plant responses that are highly controlled by different or opposing signaling pathways (Rollins et al., 2013; Johnson et al., 2014; Suzuki et al., 2014).

Heat, drought, and their combination are the main stress factors for field crops and are responsible for most production losses (Lobell et al., 2011a; Suzuki et al., 2014). Moreover, global climate change is gradually increasing the occurrence and distribution of these stressors, causing further reductions in crop yield (Rasul et al., 2011). Thus, to meet food demand, it is necessary to develop crops with elevated endurance to drought, heat stress, and their combination. Some studies have looked specifically at the effects of drought, heat stress, and their combination on barley (Rollins et al., 2013; Ashoub et al., 2015), wheat (Rampino et al., 2012; Liu et al., 2015), *Sorghum bicolor* (Johnson et al., 2014), and maize (Hu et al., 2010, 2015). However, the functions of many proteins involved in crop responses to combined drought and heat stress remain unclear.

In recent years, global quantitative analysis to determine protein expression levels has been performed using iTRAQ-based (isobaric tags for relative and absolute quantitation) methods and quantitative proteomic and LC-MS/MS (liquid chromatography/tandem mass) assays (Alvarez et al., 2014; Han et al., 2014), which facilitate the simultaneous analysis of the differential expression of proteins under control and stress conditions. Large-scale proteomic analyses have been conducted regarding crop responses to stress (Alvarez et al., 2014; Xie et al., 2016). For example, in the response of wheat to drought stress, a large number of proteins inherently exhibited different levels of expression between two varieties with different tolerances to drought stress (Alvarez et al., 2014). The genetic basis of proteome variation in crop responses to stress may represent mechanisms of stress adaptation that can be exploited in future crop-breeding efforts; this is a feasible strategy for developing drought- and heat-tolerant crop cultivars to help increase crop production under future challenging environments.

Maize (*Zea mays* L.) not only constitutes a major cereal crop, and food for both humans and animals, but has also become a critical resource for industrial use and for bio-energy production throughout the world. Maize is highly productive under suitable growth conditions. However, in many regions of the world, maize is mainly grown in semi-arid environments characterized by water scarcity, high temperature, and a combination of these conditions in the field. Maize originated from the tropics but is still sensitive to drought and heat, particularly after reaching the eight-leaf stage (Chen et al., 2010). In the maize-growing areas of China, ~60% of crops are often subjected to drought and heat, which may result in an ~30% yield loss per year. Along with global climate change, it is predicted that these stresses will become major challenges to maize yields and will lead to a loss of 15~20% of world maize production each year (Lobell et al., 2011b; Chen et al., 2012). Thus, in terms of maize breeding programs, the need to improve maize tolerance to drought, heat, and their combination has become a top priority (Chen et al., 2012).

However, at present, few studies have analyzed maize response to combined drought and heat stress at the eight-leaf stage. In this study, to discover more about such responses, we analyzed the changes in certain physical parameters and iTRAQ-based proteomes in maize exposed to heat, drought, and these conditions in combination. Furthermore, we conducted bioinformatics analyses to confirm the functions of the differentially expressed proteins in the adaptive response of maize to combined drought and heat stress. Such work should help advance our understanding of the molecular mechanisms involved in the response of maize plants to combined drought and heat stress.

## Materials and methods

### Plant material and stress treatments

According to the method we have described previously (Hu et al., 2010), maize seeds (Zhengdan 958) were used in the experiments. Zhengdan 958 is a high-yield maize hybrid that is grown in China. The seeds were surface-sterilized for 10 min in 2% hypochlorite, washed in distilled water and germinated on moistened filter paper. The maize plants were grown in Hoagland's nutrient solution in a light chamber under 400 μmol m^−2^ s^−1^ of photosynthetically active radiation, a 14-/10-h day/night cycle, a day/night temperature of 28/22°C, and a relative humidity of 75%. When the eighth leaf was fully expanded, the plants were subjected to drought, heat, and combined stress treatments.

According to our previously described procedure (Hu et al., 2010), drought stress was imposed by placing the plants in polyethylene glycol (PEG) solution (−0.7 MPa, moderate drought) for 8 h at 28°C and 40% relative humidity. Heat stress was applied by raising the temperature from 28 to 42°C at a rate of 2°C/h and then maintained at 42°C for 1 h, for a total of 8 h. Therefore, each stress treatment lasted 8 h. The combined stress consisted of simultaneous treatment with PEG and heat stress. The control seedlings were maintained at 28°C and 75% relative humidity. Next, the expanding leaves (the eighth from the bottom) of the treated and untreated seedlings were sampled, immediately frozen in liquid nitrogen, and stored at −80°C until analysis. Three biological replicates were performed for each treatment.

### Quantum efficiency of photosystem II

The quantum efficiency of photosystem II (ΦPSII) was measured using an OS-30p Chlorophyll Fluorometer (Opti-Sciences, Tyngsboro, MA, USA) on the eighth fully expanded leaf.

### Malondialdehyde (MDA) content

Malondialdehyde (MDA) content was measured according to the method described by Hodges et al. (1999): 50 mg fresh weight (FW) of leaves were homogenized in 1 ml of 80% (v/v) ethanol using a mortar and pestle. After centrifugation, the supernatant reacted with thiobarbituric acid to produce the pinkish-red chromogen, thiobarbituric acid-malondialdehyde (TBA-MDA). Absorbance was measured at 440, 532, and 600 nm by UV-vis (ultraviolet–visible) spectrophotometry. The MDA content was calculated as nmol/g FW tissue.

### Enzyme assays

According to the method we described previously (Hu et al., 2010), frozen leaf samples were homogenized (1:20 g/ml) in an extraction buffer consisting of 50 mM potassium phosphate, pH 7.0, 1 mM EDTA, and 1% polyvinylpyrrolidone, plus 1 mM ascorbate in the case of the APX assay. The homogenate was centrifuged at 15,000 × *g* for 20 min at 4°C and the supernatant was immediately used for antioxidant enzyme assays.

The activities of antioxidant enzymes were also determined by the method described previously (Hu et al., 2010). Superoxide dismutase (SOD: EC 1.15.1.1) activity was assayed by monitoring the inhibition of photochemical reduction of nitro-blue tetrazolium at 560 nm. One unit of SOD activity was defined as the amount of enzyme required to cause 50% inhibition of the nitro-blue tetrazolium reduction. APX (EC 1.11.1.11) activity was measured by monitoring the absorbance decrease at 290 nm as the ascorbate was oxidized. Glutathione reductase (GR: EC 1.6.4.2) activity was measured by following the change in oxidation at 340 nm in the glutathione-dependent oxidation of NADPH.

### Cytochemical detection of hydrogen peroxide

Hydrogen peroxide (H_2_O_2_) was visualized at the subcellular level using cerium(III) chloride (CeCl_3_) for localization (Bestwick et al., 1997; Hu et al., 2005). Electron-dense CeCl_3_ deposits are formed in the presence of H_2_O_2_ and are visible by transmission electron microscopy. Tissue pieces (1~2 mm^2^) were excised from the treated and untreated leaves and incubated in freshly prepared 5 mM CeCl_3_ in 50 mM 3-(N-morpholino) propanesulfonic acid (MOPS) at pH 7.2 for 1 h. The leaf sections were then fixed in 1.25% (v/v) glutaraldehyde and 1.25% (v/v) paraformaldehyde in 50 mM sodium cacodylate buffer, pH 7.2, for 1 h. After fixation, tissues were washed twice for 10 min in the same buffer and post-fixed for 45 min in 1% (v/v) osmium tetroxide, and then dehydrated in a graded ethanol series (30~100%; v/v) and embedded in Eponaraldite (Agar Aids, Bishop's Stortford, UK). After 12 h in pure resin, followed by a change of fresh resin for 4 h, the samples were polymerized at 60°C for 48 h. Blocks were sectioned (70~90 nm) on a Reichert-Ultracut E microtome, and mounted on uncoated copper grids (300 mesh). Sections were examined using a transmission electron microscope at an accelerating voltage of 75 kV.

### Protein extraction

As reported in our earlier study (Hu et al., 2010), total proteins from the eighth leaf of the maize plants were extracted according to the method reported by Wang et al. (2013) and Zhang et al. (2014). Briefly, ~0.5 g fresh leaves from each biological replicate were ground into a fine power in liquid nitrogen using a mortar and pestle and further ground in 4 ml of SDS buffer (30% sucrose, 2% SDS, 100 mM Tris-HCl, pH 8.0, 50 mM EDTA-Na_2_, 20 mM DTT) and 4 ml phenol (Tris-buffered, pH 8.0), then 1 mM phenylmethanesulfonyl fluoride (PMSF) and PhosSTOP phosphatase inhibitor cocktail (one tablet/10 ml; Roche, Basel, Switzerland) was added to inhibit protease and phosphatase activity. The mixture was thoroughly vortexed for 30 s and the phenol phase was separated by centrifugation at 14,000 × *g* and 4°C for 15 min. The upper phenol phase was pipetted into new 10 ml tubes, and four-fold volumes of cold methanol plus 100 mM ammonium acetate were added. After centrifugation at 14,000 × *g* and 4°C for 15 min, the supernatant was carefully discarded and the precipitated proteins were washed twice with cold acetone. Finally, the protein mixtures were harvested by centrifugation. Protein concentrations were measured using a 2-D Quant Kit (Amersham Biosciences, Piscataway, NJ, USA), with bovine serum albumin (BSA; 2 mg/ml) as the standard. To enhance the quantitative accuracy, extracted proteins from every biological replicate were adjusted to the same concentration for the subsequent analysis.

### Protein digestion and ITRAQ labeling

Protein digestion was performed according to the FASP (filter-aided sample prep) procedure described by Wiśniewski et al. (2009) and Lv et al. (2014), and the resulting peptide mixture was labeled using 4-plex iTRAQ reagent according to the manufacturer's instructions (Applied Biosystems, Foster City, CA, USA). Briefly, 200 μg of protein from each sample was mixed with 30 μl of STD buffer (4% SDS, 100 mM DTT, 150 mM Tris-HCl pH 8.0). The detergent, DTT, and other low-molecular-weight components were removed using UA buffer (8 M urea, 150 mM Tris-HCl pH 8.0) with repeated ultrafiltration (Microcon units, 30 kDa). Next, 100 μl of 0.05 M iodoacetamide in UA buffer was added to block reduced cysteine residues, and the samples were incubated for 20 min in darkness. The filters were washed three times with 100 μl of UA buffer, then twice with 100 μl of DS buffer (50 mM triethylammonium bicarbonate at pH 8.5). Finally, the protein suspensions were digested with 2 μg of trypsin (Promega, USA) in 40 μl of DS buffer overnight at 37°C, and the digested peptides were collected as a filtrate. The peptide content was estimated via UV absorption at 280 nm using an extinction coefficient of 1.1 per 0.1% (g/l) solution, which was calculated based on the proportion of tryptophan and tyrosine residues in vertebrate proteins.

For labeling, each iTRAQ reagent was dissolved in 70 μl of ethanol and added to the respective peptide mixture. The samples were referred to as control (under no stress), drought, heat, and combined drought and heat stress and were labeled with reagent and vacuum dried.

### Peptide fractionation with strong cation exchange chromatography

iTRAQ-labeled peptides were fractionated by strong cation exchange (SCX) chromatography using the AKTA Purifier system (GE Healthcare, USA). The dried peptide mixture was reconstituted and acidified with 2 ml buffer A (10 mM KH_2_PO_4_ in 25% of Acetonitrile, pH 2.7) and loaded onto a PolySULFOETHYL 4.6 × 100 mm column (5 μm, 200 Å, PolyLC Inc, MD, USA). The peptides were eluted at a flow rate of 1 ml/min with a gradient of 0–10% buffer B (500 mM KCl, 10 mM KH_2_PO_4_ in 25% of acetonitrile, pH 2.7) for 2 min, 10–20% buffer B for 25 min, 20–45% buffer B for 5 min, and 50–100% buffer B for 5 min. The elution was monitored by absorbance at 214 nm, and fractions were collected every 1 min. The collected fractions (about 30 fractions) were finally combined into 10 pools and desalted on C18 cartridges [Empore™ SPE cartridges C18 (standard density), bed I.D. 7 mm, volume 3 ml, Sigma]. Each pool was concentrated by vacuum centrifugation and reconstituted in 40 μl of 0.1% (v/v) trifluoroacetic acid. All samples were stored at −80°C until LC-MS/MS analysis.

### Liquid chromatography electrospray ionization and tandem MS (MS/MS) analysis by Q-Exactive

Analyses were performed using a Q-Exactive mass spectrometer that was coupled to an Easy-nLC system (Thermo Fisher Scientific, Odense, Denmark). Ten microliters of each fraction was injected for nanoLC-MS/MS analysis. The peptide mixture (5 μg) was loaded onto a C18 reversed-phase column (Thermo Scientific Easy Column, 10 cm long, 75 μm inner diameter, 3 μm resin) in buffer A (0.1% formic acid) and separated with a linear gradient of buffer B (80% acetonitrile and 0.1% formic acid) at a flow rate of 250 nl/min controlled by IntelliFlow technology over 140 min. MS data was acquired using a data-dependent “top10” method, which dynamically chooses the most abundant precursor ions from the survey scan (300–1800 m/z) for HCD (higher collision dissociation) fragmentation. Determination of the target value is based on predictive automatic gain control (pAGC). Dynamic exclusion duration was 60 s. Survey scans were acquired at a resolution of 70,000 at m/z 200, and resolution for HCD spectra was set to 17,500 at m/z 200. Normalized collision energy was 30 eV and the underfill ratio, which specifies the minimum percentage of the target value likely to be reached at maximum fill time, was defined as 0.1%. The instrument was run with peptide recognition mode enabled.

### Sequence database searching and data analysis

MS/MS spectra were searched using Mascot 2.2 (Matrix Science) embedded in Proteome Discoverer 1.4 against the uniprot_Zea_mays_87227_20150504.fasta (87227 sequences, downloaded on May 4, 2015) and the decoy database. For protein identification, the following options were used: peptide mass tolerance, 20 ppm; MS/MS tolerance, 0.1 Da; enzyme, trypsin; missed cleavage, 2; fixed modification Carbamidomethyl (C), iTRAQ 4-plex (K), iTRAQ 4-plex (N-term); variable modification Oxidation (M), FDR(false discovery rate) ≤ 0.01.

The protein and peptide probabilities were set at 50 and 60%, respectively. Only proteins with at least two unique peptides with a Mascot score of at least 25 and detected in at least two replicates were further analyzed.

For each replicate of proteomics, iTRAQ ratios between drought/heat/combined stress and controls for each run were converted to *z*-scores to normalize the data.

### Bioinformatics

The molecular functions of the identified proteins were classified according to their gene ontology annotations and their biological functions. The subcellular localization of the proteins identified in this study were predicted using the publicly available program WolfPsort (http://wolfpsort.org). Protein–protein interaction networks were predicted using the publicly available program STRING (http://string-db.org/). STRING is a database of known and predicted protein–protein interactions. The interactions include direct (physical) and indirect (functional) associations, and they are derived from four sources: the genomic context, high-throughput experiments, co-expression, and previous knowledge. STRING quantitatively integrates the interaction data from these sources for a large number of organisms, and where applicable, transfers information between these organisms.

According to the known or predicted cellular localization and molecular function of the proteins, as determined by Blast2Go (http://www.blast2go.com), specific groups of proteins were selected and analyzed on the basis of, for example, stimulus responses, chloroplasts proteins and enzymes.

### Statistical analysis

The mean of three replicates was used to ascertain the protein assays. Means were compared using one-way analysis of variance and Duncan's multiple range test at a 1% level of significance.

## Results

### Comparison of physical parameters affected by the three stress conditions

**Figure 1:**
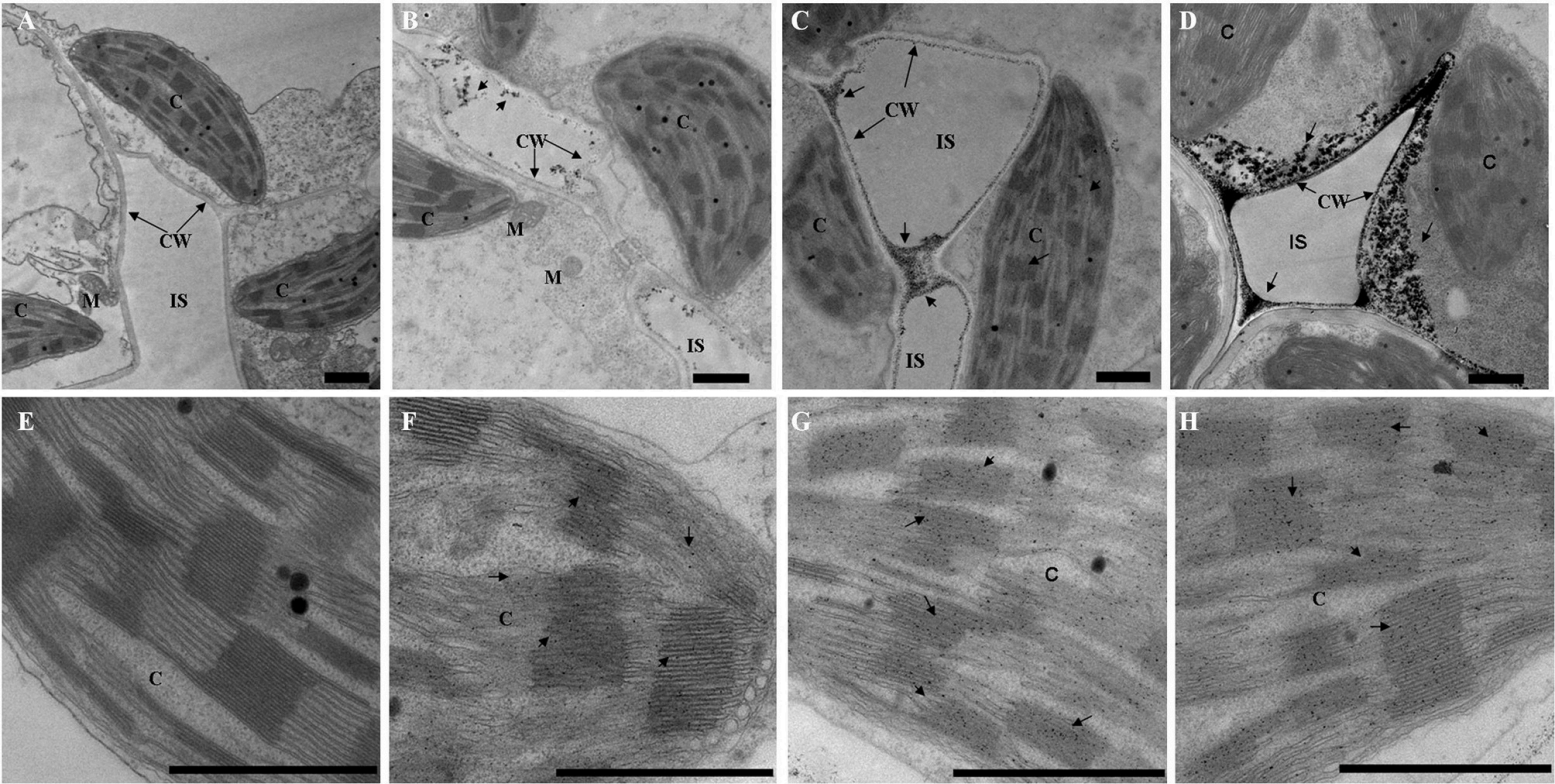
**Cytochemical localization of H_**2**_O_**2**_ accumulation in mesophyll cells of maize variety Zhengdan 958**. Arrows indicate CeCl_3_ precipitates. **(A,E)**, control; **(B,F)**, drought; **(C,G)**, heat; **(D,H)**, combined drought and heat stress; C, chloroplast; CW, cell wall; IS, intercellular space; M, mitochondrion; N, nucleus; V, vacuole. Bar = 1 μm. All experiments were repeated at least three times with similar results.

To investigate the level of H_2_O_2_ accumulation in the leaves of maize plants exposed to the drought, heat and combined stress conditions, we used a cytochemical technique whereby CeCl_3_ reacts with H_2_O_2_ to form electron-dense deposits of cerium perhydroxide (CeH_8_O_4_; Bestwick et al., 1997). Under normal conditions (control), no CeH_8_O_4_ deposit¡^a^as an indication of H_2_O_2_ accumulation¡^a^was observed in the mesophyll cells and chloroplasts (Figures 1A,E). Under the drought, heat, and combined stress conditions, H_2_O_2_ accumulation was visible in the walls of mesophyll cells (Figures 1B–D) and in chloroplasts (Figures 1F–H). Both in the walls of the mesophyll cells (Figures 1B–D) and in the chloroplasts (Figures 1F–H), the highest level of H_2_O_2_ accumulation was found under the combined stresses, and the second-highest level was observed under heat stress.

MDA is generated by lipid peroxidation, so a change in MDA content reflects the extent of membrane damage. In the present study, MDA content was prominently elevated by drought stress, heat and combined stress compared with the control (Table 1). The most obvious elevation was that under combined stress, followed by that observed under heat stress. ΦPSII is a chlorophyll fluorescence parameter that is classically used to monitor changes in photosynthetic performance. ΦPSII was significantly decreased by these three stresses. The most obvious decrease was found under the combined stress, followed by that under the heat stress.

**Table 1:** **Comparisons of physiological indexes in maize leaves under CK, D, H, and DH conditions**.

**Physiological indexes**	**CK**	**D**	**H**	**DH**
APX activity (μmol·mg^−1^ protein)	0.181d	0.218c	0.245b	0.297a
GR activity (μmol·mg^−1^ protein)	0.005d	0.006c	0.008b	0.011a
SOD activity (U·mg^−1^ protein)	11.959d	15.748c	18.807b	23.731a
MDA (nmol·g^−1^ FW)	5.120d	7.430c	9.820b	16.600a
ΦPSII	0.712a	0.610b	0.579bc	0.475c

*CK, control; D, drought stress; H, heat stress; DH, combined drought and heat stress. Each value represents the average of three biological replicas. For Duncan's Results, different characters are considered to be significant among different treatments*.

SOD catalyzes the dismutation of ${\text{O}}_{2}^{-}$ to O_2_ and H_2_O_2_. APX and GR are the two key enzymes of the Halliwell–Asada pathway for the removal of H_2_O_2_. Compared with the control, the drought, heat, and combined stress conditions enhanced the activities of SOD, APX, and GR. The most obvious elevation was under the combined stress, followed by that under the heat stress (Table 1). Taken together, these results indicate that the combined drought and heat stress had the most significant effect on these parameters, followed by the heat stress.

### Identification of differentially expressed proteins under the three stress conditions

**Figure 2:**
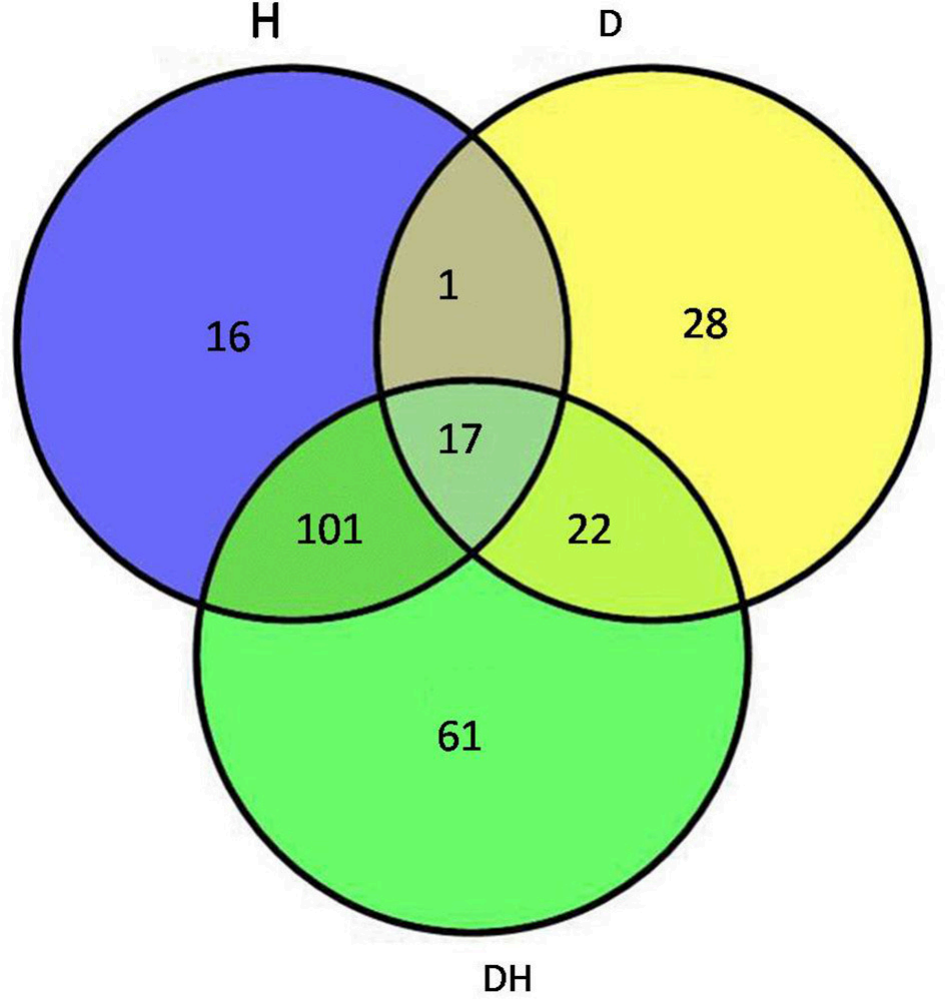
**Venn diagram showing the number of proteins with significant expression changes in maize leaves exposed to drought (D), heat (H), and combined drought and heat stress (DH)**. The diagram shows the overlap between the results from D, H, and DH.

After the maize plants at the eight-leaf stage were subjected to the drought, heat and combined stress conditions, newly expanded leaves were used to extract the total proteins, and then multiplex iTRAQ-based quantitative proteomic and LC-MS/MS assays were performed on the total proteins, resulting in the identification of 5238 proteins in these treatments at a false discovery rate (FDR) of 1%. In detail, based on a significant linear regression (*p* < 0.01) and a threshold of ≥ 1.5-fold or ≤ 0.66-fold change ratio of stress-induced protein expression levels compared with control: under the heat stress, the expression level of 135 proteins showed significant changes, of which 67 were up-regulated and 68 were down-regulated; under the drought stress, the expression level of 68 proteins showed significant changes, of which 46 were up-regulated and 22 were down-regulated; and under the combined stress, the expression level of 201 proteins showed significant changes, of which 113 were up-regulated and 88 were down-regulated (Figure 2). Among 246 proteins that showed prominent changes, 18 were commonly found under all three stress conditions (Table 1), 104 proteins were common to the heat stress and combined stress conditions (Table S1), 21 were common to the drought stress and combined stress conditions (Table S2), and one was common to the drought stress and heat stress (Table S3), while 15 proteins were identified under the heat stress alone (Table S4), 28 proteins were identified under the drought stress alone (Table S5), and 59 proteins were exclusively identified under the combined stress (Table S6).

### Proteins related to stimulus response under the three stress conditions

In this study, the expression level of 19, 39, and 59 proteins related to stimulus response showed significant changes under the drought, heat, and combined stress conditions, respectively (**Table 3**). Under all three stress conditions, ribonucleoprotein A and fatty acid desaturase were down-regulated in common (Table 1). Under the drought stress and combined stress conditions (Table S2), RAB17 protein, MTN3, uncharacterized protein (B4G1H1), glutathione S-transferase GST6, dehydrin, ABA-responsive protein and aquaporin PIP2-6 were down-regulated in common, and with the exception of MTN3, the other six proteins were significantly up-regulated. Under the heat stress and combined stress conditions (**Table 3**), 36 proteins were up-regulated in common, of which 20 were shock proteins (HSPs), including 14 small HSPs (sHSPs). All of these HSPs were obviously up-regulated by the heat stress and combined stress, but were only slightly affected by the drought stress (Table S1). In addition, 9 and 13 proteins related to stimulus response were found to be differentially expressed only under the drought stress and combined stress, respectively (**Table 3**). Of particular note was the finding that the expression levels of abscisic acid (ABA) stress ripening protein 2, ethylene-responsive protein, and ABA-, stress- and ripening-inducible-like protein were significantly up-regulated under the combined stress (Table S6). All of these proteins related to stimulus response under heat stress were found to be differentially expressed under the combined stress (**Table 3**).

### Chloroplast proteins showing significant changes

The chloroplast proteome of photosynthetic plants includes ~3000 different proteins, of which components of the photosynthetic apparatus are very abundant. In this study, 13, 21, and 32 chloroplast proteins were identified under the drought, heat and combined stress conditions, respectively. Moreover, most of the chloroplast proteins found were identified as uncharacterized proteins with molecular functions relating to nucleotide binding or catalytic activity. Under the three stress conditions (Table 2), ribonucleoprotein A, putative uncharacterized protein (B6UCG5) and uncharacterized protein (K7U7W9) were down-regulated in common. Under the heat stress and combined stress conditions (Table S1), except for iron-sulfur assembly protein IscA, the other chloroplast proteins were down-regulated in common. Eight, one and 11 chloroplast proteins were specific to the drought, heat, and combined stress conditions, respectively (Table 3). These results showed that heat and combined stress conditions may exert a more obvious effect on maize chloroplast function than drought stress.

**Table 2:** **Proteins with significant expression changes under D, H, and DH**.

**Accession**	**Description**	**D/CK**	**H/CK**	**DH/CK**	**Duncan's Results**
		**Mean (±SD)**	**Mean (±SD)**	**Mean (±SD)**	**D, H, DH**
B4F7X5	Uncharacterized protein	1.583 ± 0.120	1.500 ± 0.061	2.069 ± 0.069	b, b, a
B4FT63	Uncharacterized protein	0.653 ± 0.040	0.578 ± 0.035	0.500 ± 0.017	c, b, a
B5U8J8	Asparagine synthetase	5.280 ± 0.265	2.726 ± 0.026	10.722 ± 0.918	b, c, a
B6SID7	Late embryogenesis abundant protein, group 3	2.406 ± 0.203	1.922 ± 0.087	2.750 ± 0.260	a, b, a
B6SMU2	Putative uncharacterized protein	0.576 ± 0.030	0.651 ± 0.040	0.515 ± 0.013	b, a, c
B6SQF4	Alpha-galactosidase	0.233 ± 0.022	0.158 ± 0.019	0.133 ± 0.009	a, b, b
B6SWZ1	Sugar carrier protein C	0.446 ± 0.028	0.348 ± 0.019	0.371 ± 0.017	a, b, b
B6T531	Ribonucleoprotein A	0.639 ± 0.020	0.380 ± 0.000	0.276 ± 0.017	a, b, c
B6TEH8	Anthocyanidin 5,3-O-glucosyltransferase	0.515 ± 0.014	0.421 ± 0.018	0.405 ± 0.005	a, b, b
B6U471	Ribonucleoprotein A	0.639 ± 0.015	0.209 ± 0.009	0.184 ± 0.018	a, b, b
B6UAN2	Putative uncharacterized protein	0.578 ± 0.026	0.449 ± 0.026	0.425 ± 0.005	a, b, b
B6UCG5	Putative uncharacterized protein	0.499 ± 0.010	0.434 ± 0.018	0.437 ± 0.009	a, b, b
C0PN61	Uncharacterized protein	0.518 ± 0.018	0.556 ± 0.026	0.508 ± 0.008	b, a, b
C4J3S1	Uncharacterized protein	0.465 ± 0.023	0.389 ± 0.010	0.384 ± 0.012	a, b, b
K7U7W9	Uncharacterized protein	0.631 ± 0.018	0.465 ± 0.010	0.422 ± 0.002	a, b, c
K7UFK0	Uncharacterized protein	2.496 ± 0.008	2.402 ± 0.003	4.913 ± 0.020	b, c, a
O24626	Fatty acid desaturase (Fragment)	0.557 ± 0.024	0.552 ± 0.008	0.382 ± 0.017	a, a, b
Q42376	Late embryogenesis abundant protein, group 3	3.407 ± 0.361	1.727 ± 0.027	5.581 ± 0.010	b, c, a

*CK, control; D, drought stress; H, heat stress; DH, combined drought and heat stress. Each ratio was the average of three replicates. a Each value represents the average of three biological replicas. For Duncan's Results, different characters are considered to be significant between different treatments*.

**Table 3:** **Stimulus response proteins, chloroplast proteins and enzymes with significant expression changes under D, H, and DT respectively**.

**Response to stimulus**				**Chloroplast proteins**				**Enzymes**			
**Protein name/Protein Group Accessions**	**D**	**H**	**DH**	**Protein name/Protein Group Accessions**	**D**	**H**	**DH**	**Protein name/Protein Group Accessions**	**D**	**H**	**DH**
Ribonucleoprotein A/B6T531(B6U471)	+	+	+	Ribonucleoprotein A/B6T531	+	+	+	Alpha-galactosidase/B6SQF4	+	+	+
Fatty acid desaturase (Fragment)/O24626	+	+	+	Ribonucleoprotein A/B6U471	+	+	+	Fatty acid desaturase (Fragment)/O24626	+	+	+
RAB17 protein/A3KLI0	+	−	+	Putative uncharacterized protein/B6UCG5	+	+	+	Asparagine synthetase/B5U8J8	+	+	+
MTN3/B4FTL9	+	−	+	Uncharacterized protein/K7U7W9	+	+	+	O-succinylhomoserine sulfhydrylase/B6UAU8	+	−	+
Uncharacterized protein/B4G1H1	+	−	+	Photosystem I reaction center subunit V/B4G1K9	+	−	+	Brassinosteroid LRR receptor kinase/B6SV61	−	+	+
HVA22-like protein e/B6SRB1	+	−	+	Uncharacterized protein/B4FDE5	−	+	+	Protein kinase Kelch repeat:Kelch/B6SJR4	−	+	+
Glutathione S-transferase GSTU6/B6TLM5	+	−	+	Retinol dehydrogenase 14/B4FKX6	−	+	+	Purple acid phosphatase/B6UE38	−	+	+
Dehydrin/C4J477	+	−	+	Uncharacterized protein/B4FL89	−	+	+	Peptidyl-prolyl cis-trans isomerase/B6SRE7	−	+	+
ABA-responsive protein/K7TFB6	+	−	+	Dihydroneopterin aldolase/B4FPQ2	−	+	+	3-ketoacyl-CoA synthase/K7VSC9	−	+	+
Aquaporin PIP2-6/Q9ATM5	+	−	+	Uncharacterized protein/B4G1V3	−	+	+	Glutamyl-tRNA reductase/K7TP06	−	+	+
17.4 kDa class I heat shock protein 3/B4F976	−	+	+	Uncharacterized protein/B6SP43	−	+	+	Terpene synthase 7/Q5GJ59	−	+	+
Uncharacterized protein (Belongs to HSP20 family)/B4F9E8	−	+	+	Putative uncharacterized protein/B6SZA8	−	+	+	Exhydrolase II/Q9XE93	−	+	+
Uncharacterized protein (Blongs to HSP20 family)/B4F9K4	−	+	+	Uncharacterized protein/B6T3D8	−	+	+	Ribose-5-phosphate isomerase/B6U2Y8	−	+	+
Calcyclin-binding protein/B4FGY0	−	+	+	Ribonucleoprotein/B6TMQ1	−	+	+	Cysteine protease 1/B6TYT3	−	+	+
Uncharacterized protein/B4FL89	−	+	+	Putative uncharacterized protein/B6UAI5	−	+	+	Dihydroneopterin aldolase/B4FPQ2	−	+	+
Uncharacterized protein (HSP20 family)/B4FQS7	−	+	+	Uncharacterized protein/B8A0I4	−	+	+	PDIL1-4-*Zea mays* protein disulfide isomerase/B6UDP0	−	+	+
DnaJ subfamily B member 5 (HSP40)/B4FT54	−	+	+	Uncharacterized protein/C0P5X6	−	+	+	Retinol dehydrogenase 14/B4FKX6	−	+	+
Uncharacterized protein/B4FX40	−	+	+	Uncharacterized protein/C0P8F7	−	+	+	NADH-ubiquinone oxidoreductase chain 5 (Fragment)/Q36284	−	+	+
Uncharacterized protein/B4G1V3	−	+	+	Heat-shock protein 101/C0PDC7	−	+	+	Phosphatidate cytidylyltransferase/B4FI16	−	+	+
Uncharacterized protein (HSP20 family)/B4G250	−	+	+	Heat shock protein HSP101/Q6RYQ7	−	+	+	Extracellular ribonuclease LE/B6SSH9	−	+	+
Heat shock 70 kDa protein 1/B6SXY0	−	+	+	Uncharacterized protein/B4F9W3	+	−	−	Peptidyl-prolyl isomerase/B6TI78	−	+	+
16.9 kDa class I heat shock protein 1/B6T2J9	−	+	+	Thiamine thiazole synthase/B4FSE1	+	−	−	Peptidyl-prolyl isomerase/B6U100	−	+	+
Heat shock 22 kDa protein/B6T649	−	+	+	Uncharacterized protein/B4G206	+	−	−	Prostaglandin E synthase 3/B4FLE3	−	+	+
Phosphosulfolactate synthase-related protein/B6THJ5	−	+	+	Stachyose synthase/B6SYY2	+	−	−	Phosphosulfolactate synthase-related protein/B6THJ5	−	+	+
17.5 kDa class II heat shock protein/B6TIP9	−	+	+	Uncharacterized protein/C0P4N4	+	−	−	Peptidase, M50 family/B6UET0	−	+	+
17.4 kDa class I heat shock protein 3/B6TLK8	−	+	+	Uncharacterized protein/C0PLS3	+	−	−	Putative glycogen synthase kinase family protein/K7UT58	+	−	−
17.4 kDa class I heat shock protein 3/B6TQD6	−	+	+	Photosystem II reaction center protein L/P60138	+	−	−	Protein phosphatase 2C/B6T9X8	+	−	−
Putative uncharacterized protein/B6TQX0	−	+	+	Cytochrome b559 subunit beta/P69523	+	−	−	Thiamine thiazole synthase, chloroplastic/B4FSE1	+	−	−
16.9 kDa class I heat shock protein 1/B6TTC8	−	+	+	Iron-sulfur assembly protein IscA, mRNA/K7VC62	−	+	−	3-oxo-5-alpha-steroid 4-dehydrogenase 2/B6SU65	+	−	−
Glycine-rich RNA-binding protein 2/B6TY06	−	+	+	FtsH6-*Zea mays* FtsH protease/B4F988	−	−	+	Stachyose synthase/B6SYY2	+	−	−
Purple acid phosphatase/B6UE38	−	+	+	Uncharacterized protein/B4FHM6	−	−	+	Dihydrolipoyllysine-residue succinyltransferase component of 2-oxoglutarate dehydrogenase complex/B6TRW8	+	−	−
Peptidase, M50 family/B6UET0	−	+	+	Uncharacterized protein/B4FZN7	−	−	+	Delta 1-pyrroline-5-carboxylate synthetase/B6SKV1	+	−	−
Putative uncharacterized protein/B6UHH1	−	+	+	Tubulin alpha-6 chain/B6SR73	−	−	+	Stachyose synthase/B6SRV6	+	−	−
Small heat-shock protein/B7ZEQ0	−	+	+	Uncharacterized protein/B6TGK8	−	−	+	NADP-dependent oxidoreductase P2/B6SSU0	−	+	−
Uncharacterized protein/B8A0P3	−	+	+	Photosystem I reaction center subunit N/B6TXS5	−	−	+	Pectinesterase/C4J3B1	−	+	−
Putative heat shock protein 90 family protein/C0P4Q3	−	+	+	Uncharacterized protein/C0PFV7	−	−	+	NADH-ubiquinone oxidoreductase chain 4/Q1KK93	−	+	−
Uncharacterized protein/C0P5X6	−	+	+	Uncharacterized protein/K7U346	−	−	+	Ankyrin protein kinase-like/B6UBQ9	−	−	+
Uncharacterized protein/C0P732	−	+	+	Uncharacterized protein/K7USR3	−	−	+	Protein kinase/B6SSB7	−	−	+
Heat-shock protein 101/C0PDC7	−	+	+	Uncharacterized protein/K7UWZ6	−	−	+	Nitrate reductase [NADH] (Fragment)/P17571	−	−	+
ERTC/E1U816	−	+	+	Uncharacterized protein/K7VF90	−	−	+	Putative DEAD-box ATP-dependent RNA helicase family protein/K7VQU8	−	−	+
Uncharacterized protein/K7V2K6	−	+	+					Indole-3-acetate beta-glucosyltransferase/B6TB13	−	−	+
Uncharacterized protein (HSP70)/K7VJF3	−	+	+					3-isopropylmalate dehydrogenase/B6TJM1	−	−	+
Uncharacterized protein (Fragment)/K7VZF7	−	+	+					Aspartate aminotransferase/B4FUH2	−	−	+
Heat shock protein 82/Q08277	−	+	+					Stachyose synthase/B6UBW7	−	−	+
Heat shock protein 17.2/Q43701	−	+	+					Glutathione transferase/O24595	−	−	+
Heat shock protein HSP101/Q6RYQ7	−	+	+					Inositol-3-phosphate synthase/Q9FPK7	−	−	+
Thiamine thiazole synthase/B4FSE1	+	−	−					Phosphoethanolamine N-methyltransferase/B6T8R8	−	−	+
Delta 1-pyrroline-5-carboxylate synthetase/B6SKV1	+	−	−					Asparagine synthetase/B6ETR5	−	−	+
Stachyose synthase/B6SYY2	+	−	−					FtsH6-*Zea mays* FtsH protease/B4F988	−	−	+
MtN19-like protein/B6SZN0	+	−	−								
Protein phosphatase 2C/B6T9X8	+	−	−								
Uncharacterized protein /C0P496	+	−	−								
Uncharacterized protein/C0PLS3	+	−	−								
Uncharacterized protein/C0P4N4	+	−	−								
FtsH6-*Zea mays* FtsH protease/B4F988	−	−	+								
Abscisic stress ripening protein 2/B4FKG5	−	−	+								
Aspartate aminotransferase/B4FUH2	−	−	+								
Ethylene-responsive protein/B6T3Q3	−	−	+								
Stress protein/B6TIK3	−	−	+								
Armadillo/beta-catenin-like repeat family protein/B6TK50	−	−	+								
Uncharacterized protein (HSP70)/C4JBB8	−	−	+								
ABA-, stress-and fruit-ripening inducible-like protein/D1MN58	−	−	+								
Putative DEAD-box ATP-dependent RNA helicase family protein/K7VQU8	−	−	+								
Glutathione transferase/O24595	−	−	+								
PRm 3/P93518	−	−	+								
Inositol-3-phosphate synthase/Q9FPK7	−	−	+								

*D, drought stress; H, heat stress; DH, combined drought and heat stress. “+” or “−“ indicates common proteins or non-common proteins*.

### Responses of kinases and phosphatases to the three stress conditions

It is also notable how various enzymes (including in particular kinases and phosphatases) responded to the stresses. Under the drought stress, 12 enzymes, including one kinase and one phosphatase, were identified as differentially expressed. Under the heat stress, 27 enzymes, including two kinases and one phosphatase, were identified. Under the combined stress, 38 enzymes, including four kinases and one phosphatase, were identified (Table 3). In addition, alpha-galactosidase, fatty acid desaturase, and asparagine synthetase (B5U8J8) were commonly found under all three stress conditions. In particular, the expression level of asparagine synthetase (B5U8J8) had a 5.28- and 10.72-fold increase under the drought stress and combined stress compared with the control (Table 2), respectively, while asparagine synthetase (B6ETR5) was significantly increased only by the combined stress (Table S6). Three isoforms of stachyose synthase (B6SYY2, B6SRV6, and B6UBW7) were identified under the drought stress and combined stress, of which B6SYY2 and B6SRV6 were significantly increased by the drought stress, while B6UBW7 was increased by the combined stress; 21 enzymes were commonly found under the heat stress and combined stress (Table 3). The remaining eight, three and 13 enzymes were found only under the drought stress, heat stress, and combined stress, respectively.

**Figure 3:**
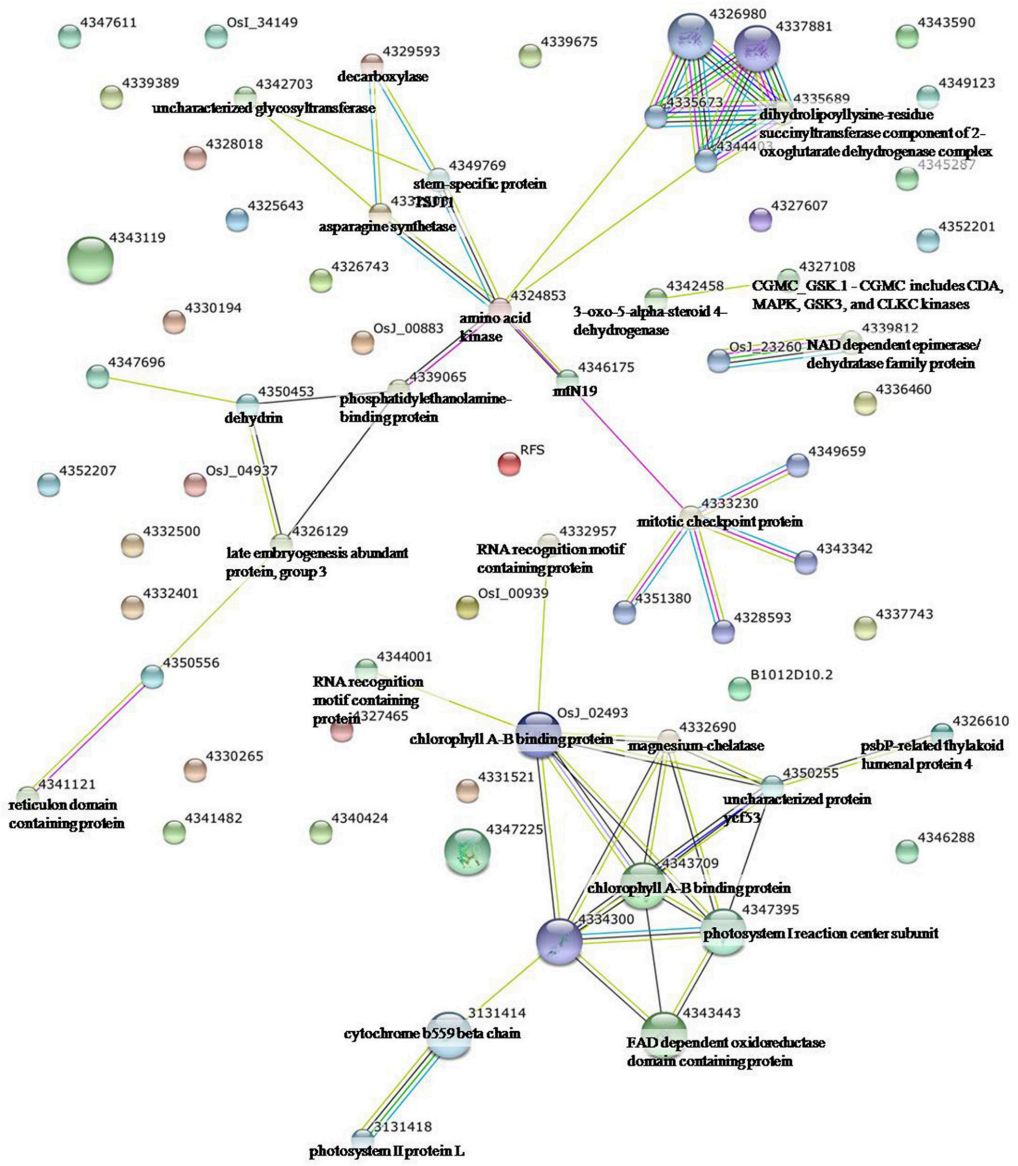
**Analysis of the protein–protein interaction network among significantly changed proteins in maize plants exposed to drought stress, using String software**.

**Figure 4:**
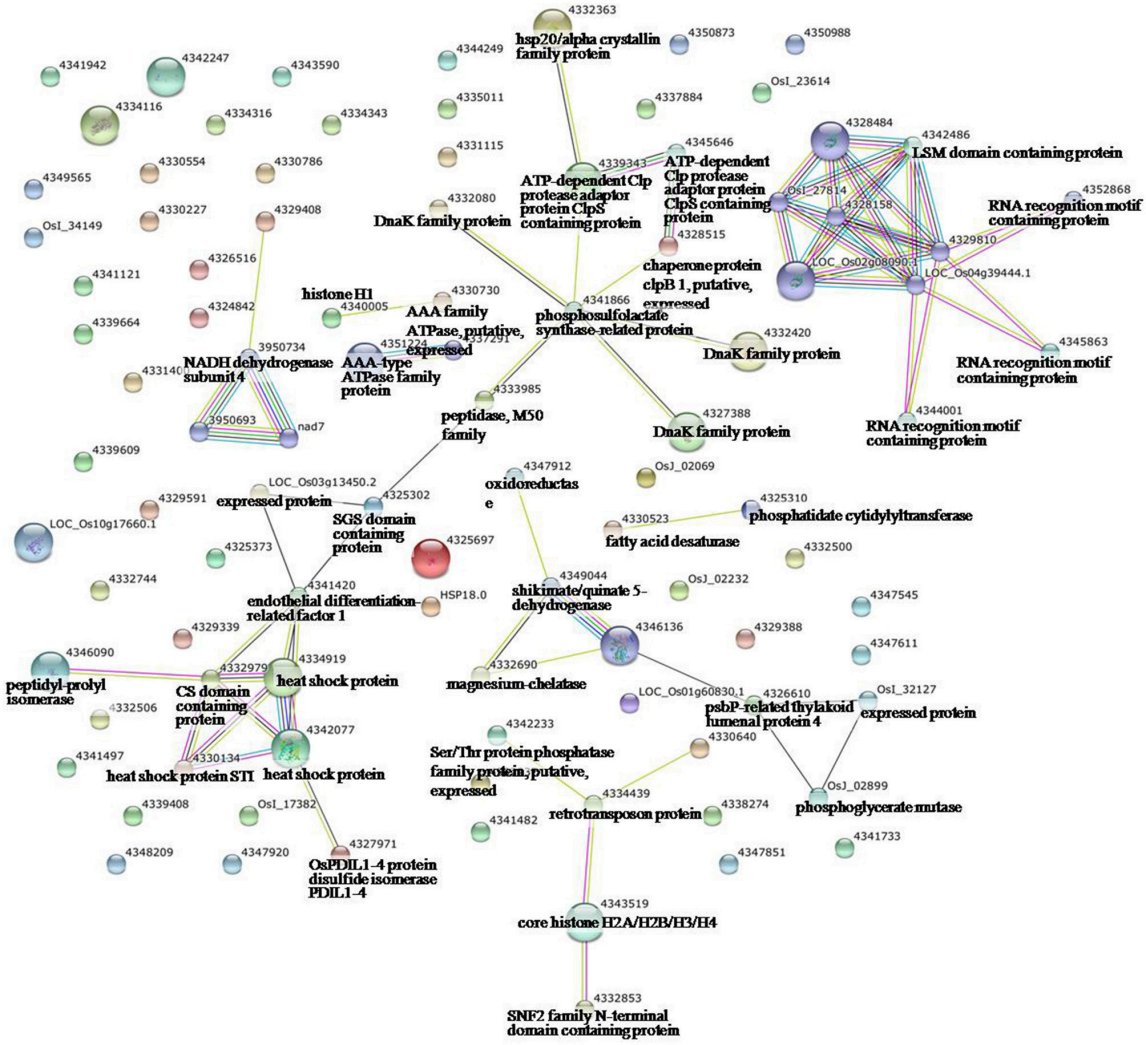
**Analysis of the protein–protein interaction network among significantly changed proteins in maize plants exposed to heat stress, using String software**.

**Figure 5:**
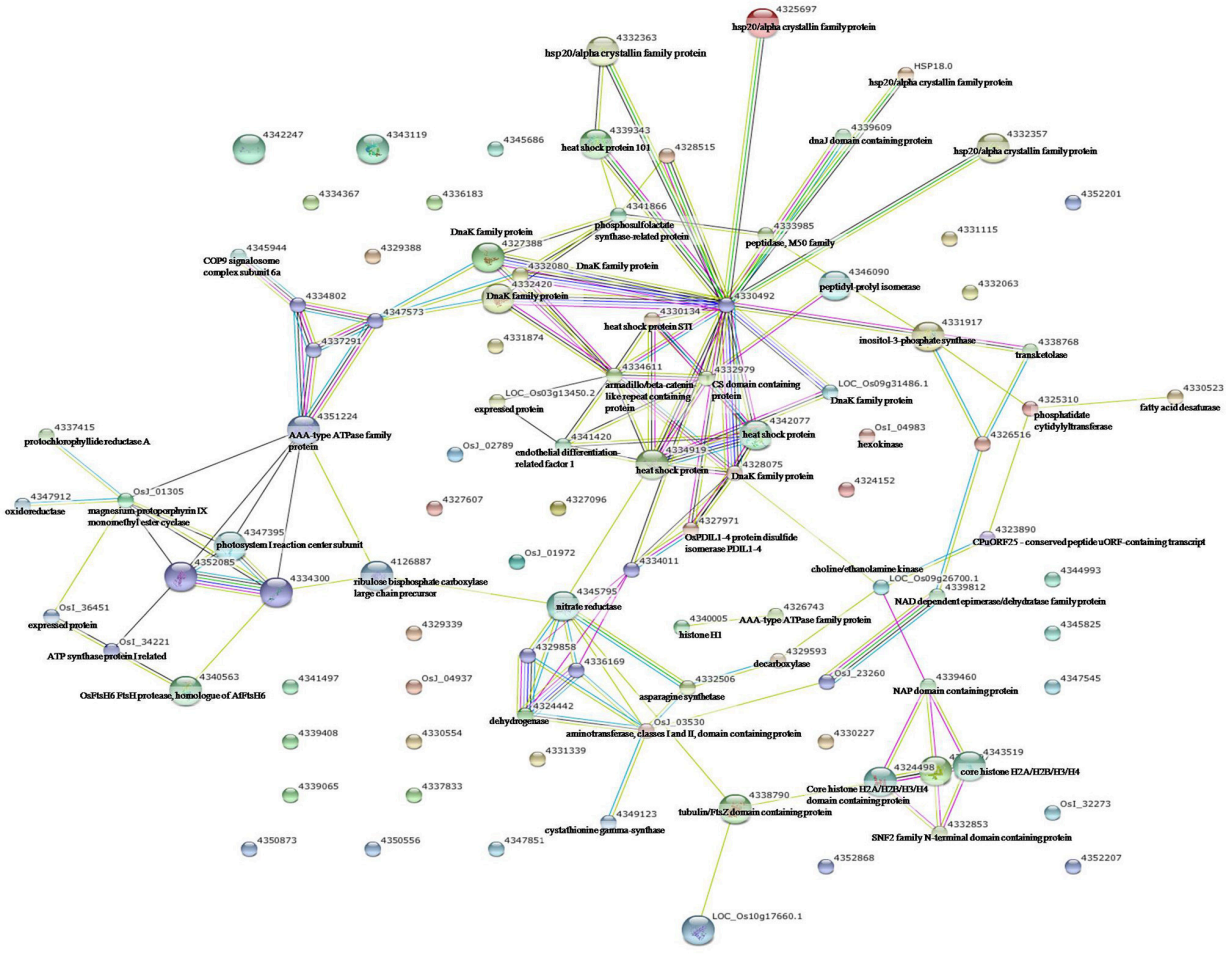
**Analysis of the protein–protein interaction network among significantly changed proteins in maize plants exposed to combined drought and heat stress, using String software**.

To identify the interactions of enzymes and HSPs with other proteins under all three stress conditions, the protein interactions among differentially expressed proteins were analyzed using STRING software (Figures 3–**5**). Under the drought stress and the combined stress, asparagine synthetase (4332506) was found to interact with decarboxylase (4329593). In fact, for all three stress conditions, extensive interactions were found among all of the chloroplast proteins (Figures 3–**5**). Under the heat stress (Figure 4) and the combined stress (Figure 5), extensive interactions were found amongst HSPs, or between HSPs and other proteins. For example, some HSPs (dnaK-family proteins 4332080, 4332420, 4327388; chaperone protein clpB1-4328515; HSP101-4339343) exhibited interactions with phosphosulfolactate synthase-related protein (4341866), while other HSPs (4334919, 4342077, 4330134) exhibited interactions with CS domain-containing protein. Interactions between HSPs were also observed under the combined stress (Figure 5). An HSP70-family protein (4330492) exhibited extensive interactions with dnaK-family proteins (4332080, 4332420, 4327388, LOC_Os09g31486.1), heat shock protein ST1 (4330134), HSP (4334919), HSP101 (4339343), HSP20/alpha crystalline family proteins (4332357, 4325697, 4332363), and HSP18 (rice protein query sequences corresponding to maize protein query sequences; see Table S7 for the drought stress, Table S8 for the heat stress, and Table S9 for the combined stress). These results indicate that HSPs as chaperones probably play a role in protecting protein functions under heat stress and combined stress conditions.

### Changes in receptor proteins

Receptors can make cells detect changes in the internal or external environment. In this study, the expression levels of three receptor proteins were significantly regulated under the drought stress, heat stress, and combined stress conditions. The expression level of brassinosteroid LRR receptor kinase (B6SV61) was reduced by the heat stress and combined stress (Table S1). The expression level of mitochondrial import receptor subunit TOM22 (B6U2X6) was increased by all three stress conditions, but under the combined stress alone there was an increase of up to 1.5-fold (Table S6). The expression level of gibberellin receptor GID1L2 (B6TC25) was decreased by the heat stress and combined stress, but under the combined stress alone there was a decrease of up to 1.5-fold (Table S6).

### The signaling pathways related to differentially expressed proteins under the three stress conditions

**Figure 6:**
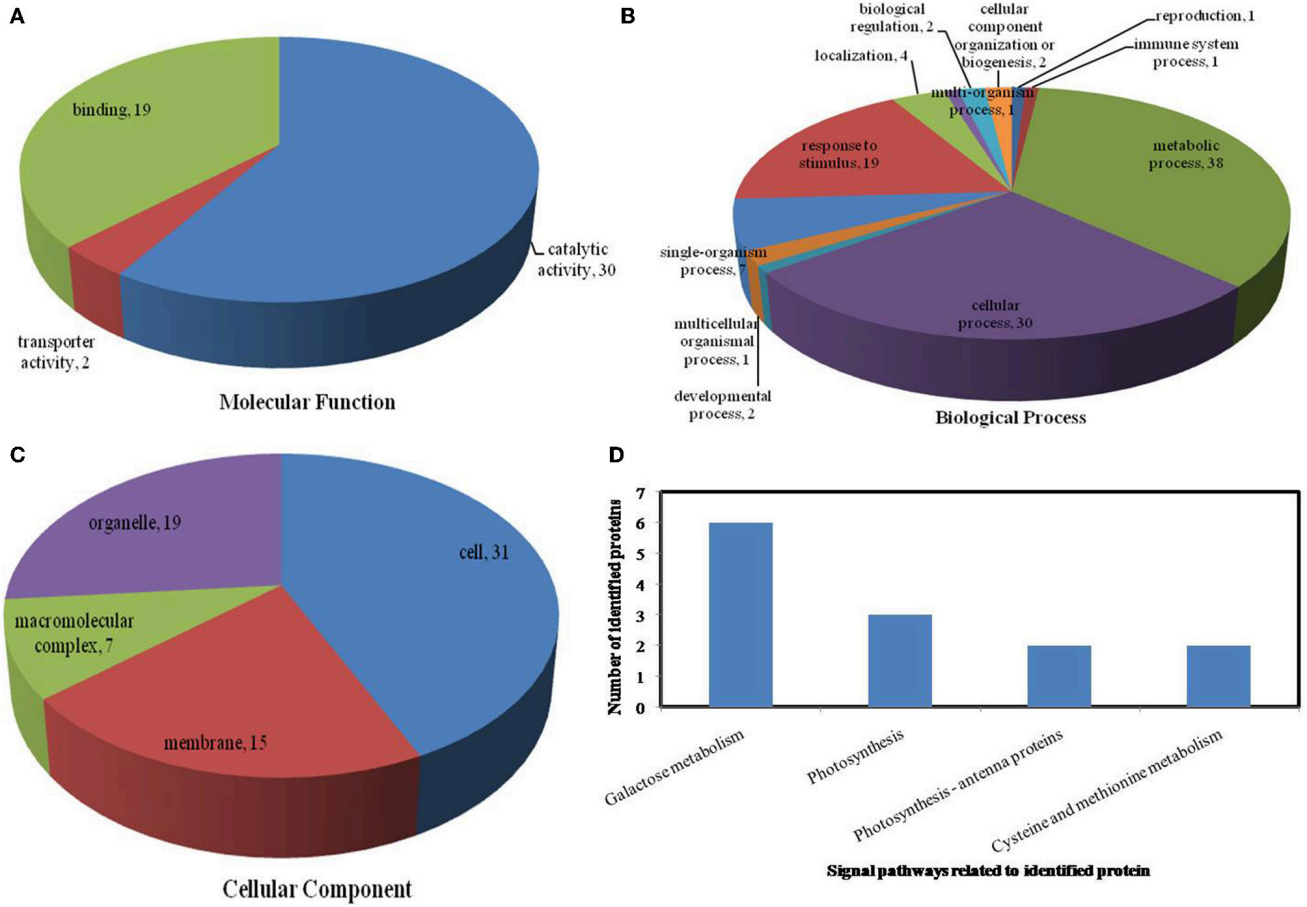
**Pie charts showing the distribution of differentially expressed proteins based on their predicted molecular functions (A), biological process (B), and cellular components (C), and the signaling pathways (D) related to differentially expressed proteins under drought stress**. Under drought stress, 65 differentially expressed proteins were identified in this study and classified according to their known or predicted cellular localization using Blast2Go (http://www.blast2go.com).

**Figure 7:**
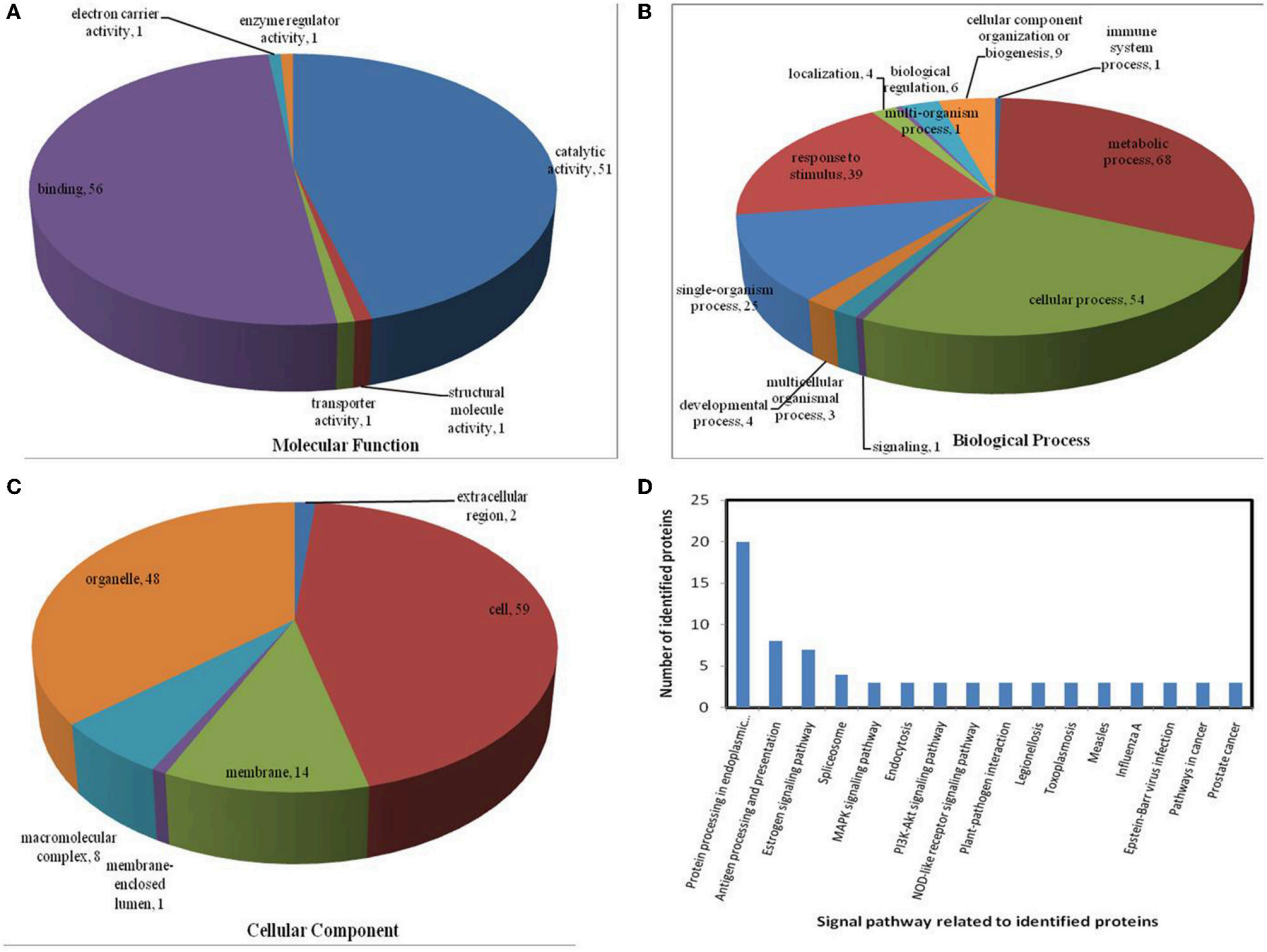
**Pie charts showing the distribution of differentially expressed proteins based on their predicted molecular functions (A), biological process (B), and cellular components (C), and the signaling pathways (D) related to differentially expressed proteins under heat stress**. Under heat stress, 135 differentially expressed proteins were identified in this study and classified according to their known or predicted cellular localization, using the Blast2Go (http://www.blast2go.com) program.

**Figure 8:**
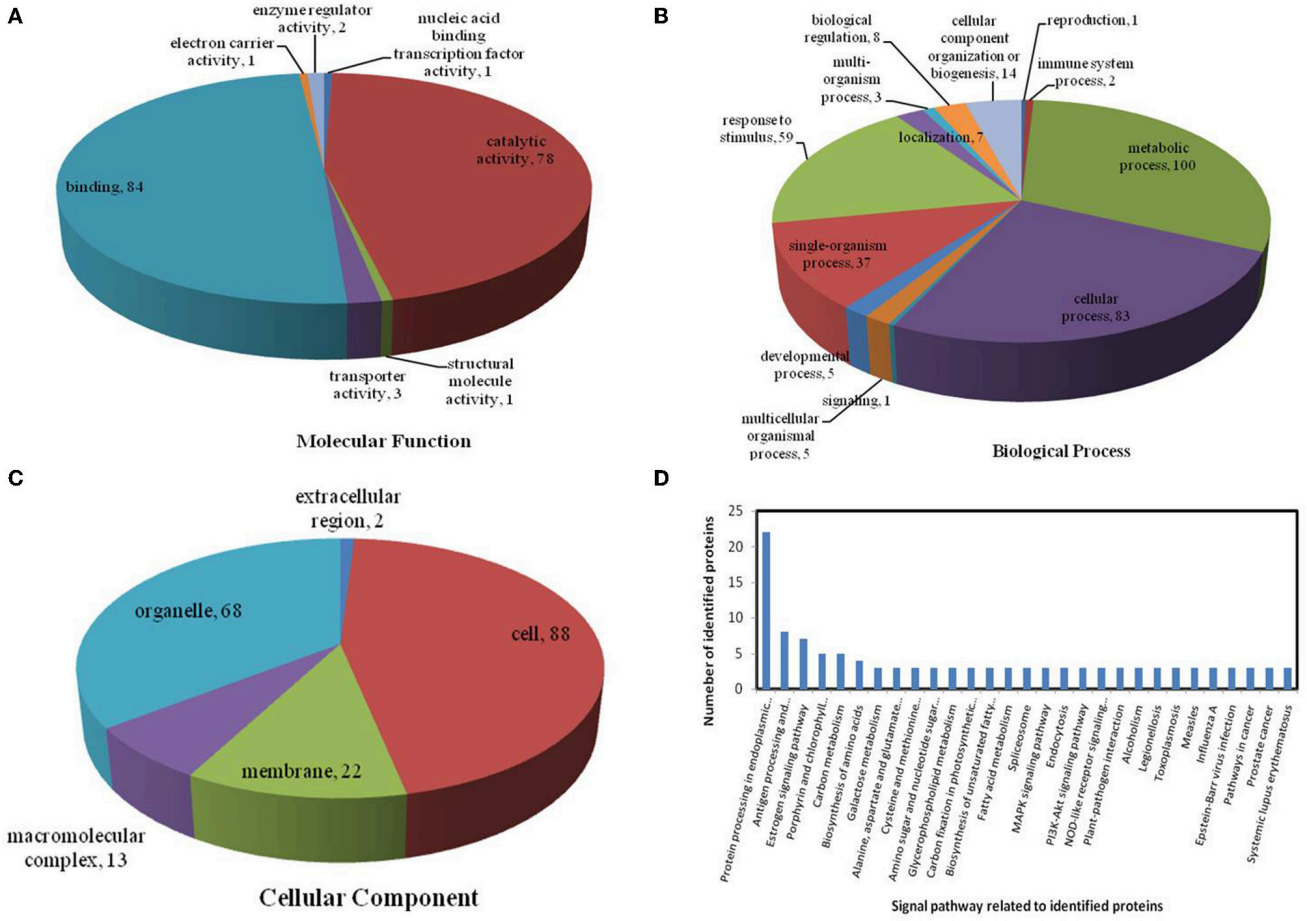
**Pie charts showing the distribution of differentially expressed proteins based on their predicted molecular functions (A), biological process (B), and cellular components (C), and the signaling pathways (D) related to differentially expressed proteins under combined drought and heat stress**. Under combined drought and heat stress, 201 proteins were identified in this study and classified according to their known or predicted cellular localization, using the Blast2Go (http://www.blast2go.com) program.

All identified proteins were classified by gene ontology (GO) annotation software and then classified as three functional groups: molecular function, biological process, and cellular component. The results of the GO analyses for the drought, heat and combined stress conditions are shown in Figures 6–**8**, respectively. Most of the annotated molecular functions were found to relate to binding and catalytic activity, while most of the annotated biological processes were found to relate to cellular and metabolic processes.

On the basis of biological process analysis using the BLAST2GO program, among differentially expressed proteins: for the drought stress, 19 proteins were classified as “response to stimulus,” two were involved in transport, and 11 were classified as binding proteins involved in DNA binding, protein binding and nucleotide binding (Figures 6A,B; Table 2); for the heat stress, 39 proteins were categorized as “response to stimulus,” one was involved in transport, and 56 were classified as binding proteins involved DNA binding, protein binding, and nucleotide binding (Figures 7A,B; Table 2); for the combined stress, 59 proteins were categorized as “response to stimulus,” three were involved in transport, and 84 were classified as binding proteins involved in DNA binding, protein binding, and nucleotide binding (Figures 8A,B; Table 2).

In the light of KEGG (Kyoto Encyclopedia of Genes and Genomes) analysis: under the drought stress, the differentially expressed proteins were found to be mainly involved in the galactose metabolism, photosynthesis, and carbon metabolism pathways (Figure 6D); under the heat stress, the differentially expressed proteins were found to be mainly involved in protein processing in the endoplasmic reticulum (ER), in antigen processing and presentation, and in estrogen signaling pathways (Figure 7D); under the combined stress, the signaling pathways were found to be similar to those found under the heat stress alone (Figure 8D). These results indicate that the signaling pathways mediated by the heat stress and combined stress were obviously different to those mediated by the drought stress. In particular, of the differentially expressed proteins that related to protein processing in endoplasmic reticulum, 20 were observed under the heat stress and 22 under the combined stress, indicating that the signaling pathways related to protein processing play an important role in maize response to heat stress and combined drought and heat stress conditions.

## Discussion

The final physiological response is dictated by the growth stage and plant tissue type, along with the severity and duration of the stress exerted on the plants. After developing to the eight-leaf stage, maize is sensitive to heat stress, especially to combined drought, and heat stress. In this study, we measured the changes in physical parameters and comprehensively analyzed the differentially expressed proteins in maize leaves in response to drought, heat, and their combination using iTRAQ-based quantitative proteomic and LC-MS/MS methods. The combined stress caused very significant changes in the level of protein expression in the maize leaf, and some changes exclusively resulted from the combined drought and heat stress.

### Physiological parameters affected by stress

The generation of reactive oxygen species (ROS) often leads to the destruction of cellular structures, which ultimately causes cell death. MDA is widely used as a marker of oxidative lipid injury. In this study, the accumulation of H_2_O_2_ and MDA, and the activities of SOD, APX, and GR, were enhanced by these three stress treatments, especially by the heat stress and combined stress. Our results indicated that all three stress conditions induced and aggravated membrane injury; in addition, they showed that the maize plants triggered an anti-oxidative defense mechanism to alleviate the ROS, which enhanced their tolerance to stress.

### Chloroplast proteins affected by combined stress

Abiotic stresses bring about serious damage to plant photosynthetic systems. In photosynthetic systems, photosystem II (ΦPSII) is one of the most sensitive components under drought and heat stresses. In soybean, 25 differentially expressed proteins of photosynthesis were involved in RuBisCO regulation, electron transport, the Calvin cycle, and carbon fixation under drought and heat stress conditions (Das et al., 2016). In our study, 13, 21, and 32 proteins were related to chloroplast function under the drought, heat, and combined stress conditions, respectively. Remarkably, under the combined stress, the 32 chloroplast proteins were mainly found to be involved in chlorophyll biosynthesis, electron transport, carbon fixation, transcription regulation, lipid metabolism, and chaperone function.

Four uncharacterized proteins (B4FHM6, B4FZN7, K7USR3, and K7UWZ6) related to chlorophyll syntheses were down-regulated, while PSI reaction center subunit V/N, HSP101, and FtsH protease were up-regulated by the heat stress and combined stress. FtsH protease is an ATP-dependent metalloprotease. In soybean, FtsH protease was up-regulated under the heat stress (Das et al., 2016). In *Arabidopsis*, FtsH protease was directly involved in turnover of the ΦPSII reaction center D1 protein (Kato et al., 2009). Taken together, our results also suggest that chloroplastic FtsHs may protect chloroplast photosynthesis under heat stress and combined stress. In rice, it was reported that ΦPSI was more susceptible to heat stress than ΦPSII (Essemine et al., 2016), which explained why the two ΦPSI reaction center subunits V and N showed significant changes under heat stress and combined stress in this study.

### Chaperone and senescence-related proteins in response to stress stimuli

Heat stress has a negative effect on protein stability and enzyme functions in the cell. Responding to this stimulus, plants synthesized HSPs and chaperone-like proteins in order to restore the correct configuration of proteins and impede aggregation (Wang et al., 2004). sHSPs play important and comprehensive roles in the ability of plants to combat heat stress (Eisenhardt, 2013; Mu et al., 2013).

In this study, protein profiles were found to be more similar under heat stress and combined stress, and were significantly different under drought stress. Among the proteins found to be differentially regulated in common under heat stress and combined stress, HSPs, including 14 sHSPs, were the most-represented. Similar results were found in terms of the response of wild barley and soybean to heat stress and combined drought and heat stress (Ashoub et al., 2015; Das et al., 2016), but more HSPs were identified in the present study, which supports our finding that maize is highly sensitive to heat at the eight-leaf stage. In this study, HSPs interacted strongly with other proteins or with HSPs under heat stress and combined stress. These results are the first to demonstrate the similarity of HSP expression in the response of maize to heat stress and combined drought and heat stress, and reaffirm that HSPs are important in terms of plant responses to heat stress and combined drought and heat stress.

The abundance of some proteins was exclusively changed under the combined stress. In particular, the combined stress increased the expression of ftsH6-*Z. mays* FtsH protease, which had a predicted interaction with photosystem I reaction center subunit, ATP synthase protein I. It has been reported that ATP-dependent zinc metalloprotease FTSH 1 is involved in the turnover of oxidatively damaged D1 proteins of ΦPSII (Adam et al., 2001) and contributes to the heat tolerance of grapevine (Rocheta et al., 2014). These results indicate that the distinct forms of metalloprotease FTSH may play an active role in protecting chloroplast from heat stress and combined drought and heat stress. Moreover, the expression of ABA stress ripening-related protein 2, ethylene-responsive protein, and ABA-, stress- and ripening-inducible-like protein was found to be significantly increased under all three stress conditions. These three proteins are all associated with leaf senescence and fruit ripening. In plants, leaf senescence promotes the transfer of nutrients to developing and storage tissues. It has been reported that the senescence and abscission of older leaves, and the subsequent transfer of nutrients, increases plant survival under drought and heat stress (Munné-Bosch and Alegre, 2004; Lim et al., 2007). In addition, studies on transgenic tobacco have indicated that delayed leaf senescence increases tobacco endurance to drought stress (Rivero et al., 2007). Furthermore, studies have shown that ABA affects mango fruit ripening by regulating ethylene changes (Zaharah et al., 2013), and promotes leaf senescence by enhancing ethylene production in submerged aquatic plants (Jana and Choudhuri, 1982). Nevertheless, with regard to the response of *Arabidopsis* to drought stress, the study by Zhao et al. (2016) found that ABA promotes leaf senescence in an ethylene-independent manner. However, further research is needed to further prove whether ABA and ethylene can enhance maize tolerance to drought, heat and combined stress conditions by promoting leaf senescence.

### Protein processing in the ER

The endoplasmic reticulum (ER) is an important organelle responsible for proteostasis. The accumulation of misfolded proteins in the ER disturbs ER homeostasis and thus brings about ER stress. Misfolded proteins may bind to chaperone BiP and be degraded through the proteasome (Perri et al., 2016). The protein disulfide isomerase (PDI) is an abundant oxidoreductase in eukaryotic ER and catalyzes the folding of proteins (Gruber et al., 2006). HSPs may not only prevent the inappropriate interaction of proteins and promote correct folding, but may also play a significant role in the degradation pathways (Bozaykut et al., 2014).

In this study, the protein processing that occurred in ER was the most prominent pathway under the heat stress and combined stress stimulus. In particular, we observed one down-regulated PDI and 17 up-regulated HSPs (including 12 sHSPs) under the heat stress and combined stress. The results suggest that the depression of PDI expression may cause the accumulation of misfolded proteins in ER. Thus, the expression of HSPs was significantly elevated in order to eliminate misfolded proteins. It is important to uncover the role of HSPs in protein turnover under heat stress and combined stress.

### Phosphatases and kinases

The interplay between phosphatases and kinases strictly controls many biological processes in plants (Johnson, 2009; Pjechová et al., 2014). Brassinosteroids (BRs) regulate various aspects of plant development (Yang et al., 2011). It is well-known that BR and ABA exert an antagonistic effect on plant development. BR signaling mutant *bak1* (*BRI1-associated receptor kinase 1*) has been found to lose more water than wild-type and to be insensitive to ABA in stomatal closure, suggesting that BAK1 is involved in stomatal closure induced by ABA (Shang et al., 2016). Our own results showed that heat stress and combined stress down-regulated the expression of brassinosteroid LRR receptor kinase. So, we hypothesized that brassinosteroid LRR receptor kinase may have a similar function to BAK1 under heat stress and combined stress. Namely, in relation to the control plants, the decrease in the level of BAK1 expression may have caused the maize leaf to lose more water, which would be helpful in adapting to the heat stress and combined stress conditions.

Plant glycogen synthase kinase-3 (GSK-3) belongs to a multigene family that regulates various different physiological responses (Saidi et al., 2012; Youn and Kim, 2015). It has been reported that alfalfa MsK4 and *Arabidopsis* AtGSK1/ASK1 and ASKα promote enhanced salt tolerance (Piao et al., 2001; Kempa et al., 2007; Santo et al., 2012); However, rice GSK1 has been shown to reduce salt tolerance (Koh et al., 2007). The study by Youn and Kim (2015) revealed that AtSK21/BIN2 and AtSK12 was critical in BR signaling. Our own results indicated that these three stress conditions down-regulated the expression of putative glycogen synthase kinase family protein. However, in terms of the response of maize response to drought, heat and combined stress conditions, the role of GSKs in BR and ABA signaling needs further investigation.

The expression of protein kinase Kelch repeat/Kelch was elevated by the heat stress and combined drought and heat stress. In rice, Kelch domain containing 10 has been shown to be involved in oxidative stress-induced cell death (Sekine et al., 2012), and OsFBK12 (an F-box protein containing a Kelch repeat motif) regulated pleiotropic phenotypes and leaf senescence (Chen et al., 2013). Purple acid phosphatase (PAP) family members were involved in extensive aspects of plant development, mineral homeostasis and stress responses (González-Muñoz et al., 2015). In *Arabidopsis thaliana*, the elimination of AtPAP26 disturbed phosphorus remobilization and delayed leaf senescence (Robinson et al., 2012). In the current study, heat stress and combined stress down-regulated PAP expression. However, the role of protein kinase Kelch repeat and PAPs in maize endurance to heat stress and combined stress remains to be elucidated.

### Proteins involved in K^+^, sugar, and water transport

Transporter proteins play important roles in maintaining turgor pressure and regulating water potential, which is vital for plant growth and survival in the stress response. For example, plasma membrane intrinsic proteins (PIPs) are primary channels that mediate the transfer of water and other small molecules across vacuolar and plasma membranes, and are associated with plant tolerance to stress. In this study, drought stress and combined stress up-regulated the expression of aquaporin PIP2-6. Other results have shown that the over-expression of MzPIP2:1 in *Arabidopsis* enhances plant tolerance to drought (Wang et al., 2015). However, the over-expression of AtPIP1:2 in tobacco has been found to reduce plant tolerance to drought (Aharon et al., 2003).

K^+^ is involved in many cellular processes, including enzyme activation, protein synthesis, and osmotic regulation (Anschütz et al., 2014; Demidchik et al., 2014). In *Arabidopsis* responses to drought, it is essential to regulate the homeostasis of intracellular K^+^. KZM2 has a voltage-gated K^+^ channel activity (Shabala and Pottosin, 2014). The study by Büchsenschütz et al. (2005) found that KZM2 in maize epidermis was responsible for stomatal opening. In the present study, both heat stress and combined stress up-regulated the level of KZM2 expression. Taken together, these results indicate that KZM2 enhances plant tolerance to heat stress and combined drought and heat stress by regulating stomatal opening, which helps to release heat by transpiration.

Under abiotic stress conditions, carbohydrates accumulate in plant cells. In *Arabidopsis* leaves, the carrier protein SWEET17 is a major factor controlling fructose metabolism. The decrease of SWEET17 expression by stress causes fructose to accumulate in leaves (Chardon et al., 2013). SWEET16 is a vacuole-located carrier involved in glucose, fructose, and sucrose transportation. The over-expression of AtSWEET16 has been shown to modify *Arabidopsis* tolerance to stress (Klemens et al., 2013). In this study, sugar carrier protein C had a significant decrease under these three stress conditions. Taken together, these results indicate that the reduced expression of a sugar transporter may facilitate the accumulation of sugar in leaves in order to increase stress endurance.

## Conclusions

Owing in part to climate change, food resources are being challenged by drought, heat, and the combination of these factors. Plants apply different mechanisms to adapt to combined stresses than to adapt to a single stress. Among the drought, heat, and combined drought and heat stress conditions, we found more similar proteins between the heat stress and combined stress conditions. HSPs, especially sHSPs, showed abundant expression under the heat stress and combined stress, and were found to play a role in extensive signaling pathways, suggesting that HSPs play a crucial role in maize tolerance to heat stress and combined stress. Even though similar signaling pathways were found in response to the heat stress and combined stress, relative to the drought stress and heat stress, the combined stress led to the greater expression of chloroplast proteins, enzymes and stimuli response proteins, which led to the development of more extensive signaling pathways and protein interaction networks. Our results also implied that ethylene-responsive protein and ripening-related proteins, which promote leaf senescence, may also have a potential role in maize endurance to combined drought and heat stress. Therefore, our results could be used to further our understanding of the mechanisms of crop response to combined stresses.

## Author contributions

XH conceived and designed the research. FZ, YZ, and FT performed the experiments. DZ, HY, and XH analyzed the data. WW and CL contributed reagents/materials/analysis tools. XH and DZ wrote the paper. All authors read and approved the manuscript.

### Conflict of interest statement

The authors declare that the research was conducted in the absence of any commercial or financial relationships that could be construed as a potential conflict of interest.
