# Supplementary material for: The Difference of Physiological and Proteomic Changes in Maize Leaves Adaptation to Drought, Heat, and Combined Both Stresses
Source: Front Plant Sci. 2016 Oct 26;7:1471. doi: 10.3389/fpls.2016.01471 (PMC5080359; doi:10.3389/fpls.2016.01471)
Supplement: Supplementary file 1 [file Table1.DOC]

**Table S1︱P**roteins with significant expression level changes under H and DH

| Accession | Description | D/CK | | H/CK | | DH/CK | | Duncan's Results |
| --- | --- | --- | --- | --- | --- | --- | --- | --- |
|  |  |  |  |  |  |  |  |  |
|  |  | Mean (±SD) | P-Value | Mean (±SD) | P-Value | Mean (±SD) | P-Value | D, H, DH |
| A4KA61 | Profilin | 0.711±0.011 | 0.000 | 0.406±0.009 | 0.000 | 0.413±0.017 | 0.000 | a, b, b |
| B4F8F5 | Uncharacterized protein | 0.847±0.020 | 0.006 | 0.549±0.049 | 0.004 | 0.564±0.032 | 0.002 | a, b, b |
| B4F976 | 17.4 kDa class I heat shock protein 3 | 0.757±0.024 | 0.003 | 4.662±0.469 | 0.005 | 5.374±0.303 | 0.002 | c, b, a |
| B4F9E8 | Uncharacterized protein | 0.950±0.056 | 0.260 | 1.823±0.154 | 0.011 | 2.664±0.148 | 0.004 | c, b, a |
| B4F9K4 | Uncharacterized protein | 1.029±0.010 | 0.034 | 3.244±0.042 | 0.000 | 4.650±0.377 | 0.004 | c, b, a |
| B4FA43 | Uncharacterized protein | 0.992±0.026 | 0.653 | 0.599±0.044 | 0.004 | 0.614±0.021 | 0.001 | a, b, b |
| B4FDE5 | Uncharacterized protein | 0.897±0.006 | 0.001 | 0.624±0.021 | 0.001 | 0.520±0.028 | 0.001 | a, b, c |
| B4FFS8 | Uncharacterized protein | 0.768±0.018 | 0.002 | 0.395±0.000 | 0.000 | 0.406±0.000 | 0.000 | a, b, b |
| B4FGY0 | Calcyclin-binding protein | 0.949±0.043 | 0.174 | 1.669±0.103 | 0.008 | 1.903±0.116 | 0.006 | b, a, a |
| B4FI16 | Phosphatidate cytidylyltransferase | 1.187±0.097 | 0.079 | 1.517±0.000 | 0.000 | 1.535±0.035 | 0.001 | b, a, a |
| B4FIA6 | Histone H2A | 0.922±0.021 | 0.022 | 2.019±0.104 | 0.003 | 1.931±0.000 | 0.000 | b, a, a |
| B4FKX6 | Retinol dehydrogenase 14 | 1.303±0.099 | 0.033 | 1.508±0.011 | 0.000 | 1.503±0.000 | 0.000 | b, a, a |
| B4FL89 | Uncharacterized protein | 0.910±0.095 | 0.244 | 1.527±0.022 | 0.001 | 1.621±0.155 | 0.010 | b, a, a |
| B4FLE3 | Prostaglandin E synthase 3 | 0.817±0.016 | 0.003 | 1.893±0.098 | 0.004 | 2.067±0.064 | 0.001 | c, b, a |
| B4FME3 | Uncharacterized protein | 0.775±0.022 | 0.003 | 2.069±0.104 | 0.003 | 1.627±0.074 | 0.005 | c, a, b |
| B4FPQ2 | Dihydroneopterin aldolase | 0.698±0.086 | 0.026 | 0.630±0.026 | 0.002 | 0.631±0.030 | 0.002 | a, a, a |
| B4FQS7 | Uncharacterized protein | 1.021±0.018 | 0.184 | 1.577±0.073 | 0.005 | 1.983±0.174 | 0.010 | c, b, a |
| B4FT54 | DnaJ subfamily B member 5 | 0.940±0.087 | 0.355 | 1.542±0.041 | 0.002 | 1.715±0.094 | 0.006 | c, b, a |
| B4FUV7 | Uncharacterized protein | 0.741±0.017 | 0.001 | 0.278±0.014 | 0.000 | 0.280±0.028 | 0.000 | a, b, b |
| B4FX40 | Uncharacterized protein | 1.010±0.070 | 0.828 | 0.547±0.027 | 0.001 | 0.551±0.030 | 0.001 | a, b, b |
| B4FZD7 | Uncharacterized protein | 0.837±0.023 | 0.007 | 0.640±0.066 | 0.011 | 0.530±0.014 | 0.000 | a, b, c |
| B4G1V3 | Uncharacterized protein | 0.815±0.013 | 0.002 | 0.446±0.018 | 0.000 | 0.376±0.038 | 0.001 | a, b, c |
| B4G250 | Uncharacterized protein | 0.915±0.100 | 0.279 | 3.934±0.318 | 0.004 | 4.636±0.394 | 0.004 | c, b, a |
| B6SIA6 | Uncharacterized protein | 1.152±0.051 | 0.036 | 1.634±0.089 | 0.006 | 1.691±0.087 | 0.005 | b, a, a |
| B6SI29 | Histone H2A | 1.169±0.038 | 0.017 | 1.532±0.020 | 0.002 | 1.597±0.063 | 0.004 | b, a, a |
| B6SJR4 | Protein kinase Kelch repeat:Kelch | 0.915±0.085 | 0.225 | 2.382±0.205 | 0.007 | 1.831±0.174 | 0.014 | c, a, b |
| B6SP43 | Uncharacterized protein | 0.887±0.009 | 0.002 | 1.622±0.111 | 0.011 | 1.587±0.051 | 0.003 | b, a, a |
| B6SQN7 | Putative uncharacterized protein | 1.095±0.092 | 0.216 | 1.687±0.000 | 0.000 | 1.879±0.147 | 0.009 | b, a, a |
| B6SRE7 | Peptidyl-prolyl cis-trans isomerase | 1.022±0.021 | 0.212 | 0.468±0.000 | 0.000 | 0.466±0.018 | 0.000 | a, b, b |
| B6SSH9 | Extracellular ribonuclease LE | 0.980±0.005 | 0.020 | 1.966±0.203 | 0.014 | 1.536±0.030 | 0.001 | c, a, b |
| B6STA5 | Glycine-rich RNA-binding protein 2 | 0.939±0.035 | 0.096 | 0.405±0.012 | 0.000 | 0.376±0.000 | 0.000 | a, b, b |
| B6SV61 | Brassinosteroid LRR receptor kinase | 1.156±0.099 | 0.111 | 0.590±0.061 | 0.007 | 0.542±0.000 | 0.000 | a, b, b |
| B6SXY0 | Heat shock 70 kDa protein 1 | 0.899±0.090 | 0.191 | 1.936±0.177 | 0.012 | 2.549±0.049 | 0.000 | c, b, a |
| B6SZ50 | Retrotransposon protein | 0.943±0.039 | 0.125 | 1.925±0.100 | 0.004 | 2.130±0.217 | 0.012 | b, a, a |
| B6SZA8 | Putative uncharacterized protein | 0.886±0.042 | 0.042 | 0.645±0.026 | 0.002 | 0.598±0.046 | 0.004 | b, a, a |
| B6T026 | Cortical cell-delineating protein | 0.677±0.044 | 0.006 | 0.531±0.000 | 0.000 | 0.665±0.014 | 0.001 | a, b, a |
| B6T2J9 | 16.9 kDa class I heat shock protein 1 | 0.951±0.036 | 0.143 | 2.225±0.000 | 0.000 | 2.835±0.121 | 0.001 | c, b, a |
| B6T3D8 | Uncharacterized protein | 0.944±0.061 | 0.252 | 0.608±0.087 | 0.011 | 0.525±0.019 | 0.001 | a, b, b |
| B6T630 | Putative uncharacterized protein | 1.079±0.078 | 0.220 | 0.612±0.013 | 0.000 | 0.579±0.019 | 0.001 | a, b, b |
| B6T649 | Heat shock 22 kDa protein | 0.958±0.018 | 0.054 | 3.617±0.365 | 0.006 | 4.284±0.183 | 0.001 | c, b, a |
| B6TA56 | Histone H1 | 1.048±0.040 | 0.173 | 2.100±0.195 | 0.010 | 2.130±0.191 | 0.009 | b, a, a |
| B6THJ5 | Phosphosulfolactate synthase-related protein | 0.899±0.089 | 0.187 | 2.024±0.113 | 0.004 | 2.082±0.139 | 0.005 | b, a, a |
| B6TI78 | Peptidyl-prolyl isomerase | 1.003±0.002 | 0.122 | 1.547±0.042 | 0.002 | 1.610±0.165 | 0.023 | b, a, a |
| B6TIJ3 | Thioredoxin | 0.976±0.019 | 0.156 | 1.801±0.101 | 0.005 | 1.781±0.062 | 0.002 | b, a, a |
| B6TIP9 | 17.5 kDa class II heat shock protein | 0.961±0.036 | 0.202 | 2.597±0.156 | 0.003 | 3.545±0.389 | 0.008 | c, b, a |
| B6TKI8 | Putative uncharacterized protein | 1.285±0.087 | 0.029 | 1.763±0.111 | 0.007 | 1.840±0.187 | 0.006 | b, a, a |
| B6TLK8 | 17.4 kDa class I heat shock protein 3 | 0.831±0.027 | 0.008 | 3.327±0.291 | 0.005 | 5.433±0.332 | 0.002 | c, b, a |
| B6TMB1 | Putative uncharacterized protein | 1.041±0.040 | 0.218 | 0.616±0.018 | 0.001 | 0.612±0.020 | 0.001 | a, b, b |
| B6TMQ1 | Ribonucleoprotein | 0.766±0.000 | 0.000 | 0.537±0.033 | 0.002 | 0.599±0.027 | 0.001 | a, c, b |
| B6TQD6 | 17.4 kDa class I heat shock protein 3 | 0.996±0.000 | 0.368 | 2.042±0.125 | 0.005 | 1.855±0.040 | 0.001 | c, a, b |
| B6TQG2 | Stress-related protein | 0.982±0.000 | 0.016 | 1.523±0.024 | 0.001 | 1.593±0.082 | 0.006 | b, a, a |
| B6TQX0 | Putative uncharacterized protein | 0.883±0.079 | 0.124 | 1.598±0.087 | 0.007 | 1.793±0.118 | 0.007 | c, b, a |
| B6TTC8 | 16.9 kDa class I heat shock protein 1 | 0.922±0.000 | 0.000 | 3.490±0.230 | 0.003 | 3.442±0.352 | 0.007 | b, a, a |
| B6TTV8 | WTF1 | 0.896±0.000 | 0.000 | 0.617±0.016 | 0.001 | 0.526±0.023 | 0.001 | a, b, c |
| B6TY06 | Glycine-rich RNA-binding protein | 1.295±0.087 | 0.028 | 0.454±0.040 | 0.002 | 0.412±0.020 | 0.000 | a, b, b |
| B6TYT3 | Cysteine protease 1 | 0.965±0.044 | 0.299 | 0.645±0.026 | 0.002 | 0.627±0.000 | 0.000 | a, b, b |
| B6U100 | Peptidyl-prolyl isomerase | 1.132±0.101 | 0.151 | 2.066±0.176 | 0.009 | 2.045±0.069 | 0.001 | b, a, a |
| B6U2Y8 | Ribose-5-phosphate isomerase | 1.095±0.079 | 0.171 | 0.574±0.044 | 0.003 | 0.622±0.035 | 0.003 | a, b, b |
| , bB6UAI5 | Putative uncharacterized protein | 1.051±0.007 | 0.005 | 1.774±0.173 | 0.011 | 1.784±0.139 | 0.010 | b, a, a |
| B6UDP0 | PDIL1-4-Zea mays protein disulfide isomerase | 0.883±0.079 | 0.124 | 0.650±0.087 | 0.020 | 0.633±0.052 | 0.007 | a, b, b |
| B6UE38 | Purple acid phosphatase | 0.984±0.069 | 0.725 | 0.610±0.087 | 0.006 | 0.579±0.017 | 0.001 | a, b, b |
| B6UEB0 | Lipid binding protein | 0.758±0.037 | 0.008 | 0.652±0.046 | 0.006 | 0.575±0.000 | 0.000 | a, b, c |
| B6UET0 | Peptidase, M50 family | 1.130±0.100 | 0.152 | 1.680±0.125 | 0.011 | 2.166±0.000 | 0.000 | c, a, b |
| B6UFX4 | Histone H2B | 1.052±0.038 | 0.142 | 1.564±0.020 | 0.008 | 1.547±0.063 | 0.004 | b, a, a |
| B6UHB6 | Histone H1 | 1.012±0.106 | 0.863 | 1.508±0.013 | 0.000 | 1.562±0.053 | 0.004 | b, a, a |
| B6UHH1 | Putative uncharacterized protein | 0.989±0.052 | 0.747 | 1.906±0.198 | 0.015 | 1.996±0.250 | 0.010 | b, a, a |
| B7ZEQ0 | Small heat-shock protein | 0.926±0.025 | 0.036 | 2.445±0.265 | 0.011 | 3.025±0.114 | 0.001 | c, b, a |
| B8A0I4 | Uncharacterized protein | 0.877±0.021 | 0.010 | 0.579±0.037 | 0.003 | 0.572±0.028 | 0.001 | a, b, b |
| B8A0P3 | Uncharacterized protein | 1.105±0.087 | 0.172 | 1.589±0.068 | 0.004 | 1.739±0.000 | 0.000 | c, b, a |
| B8Y6I0 | Chloroplast pentatricopeptide repeat protein 10 | 0.780±0.020 | 0.003 | 0.596±0.021 | 0.001 | 0.553±0.036 | 0.002 | a, b, b |
| C0HG21 | Uncharacterized protein | 0.912±0.087 | 0.223 | 0.485±0.066 | 0.005 | 0.576±0.026 | 0.001 | a, b, b |
| C0P4Q3 | Putative heat shock protein 90 family protein | 0.996±0.078 | 0.937 | 3.316±0.293 | 0.005 | 3.564±0.357 | 0.006 | b, a, a |
| C0P5X6 | Uncharacterized protein | 0.989±0.061 | 0.784 | 1.951±0.092 | 0.009 | 2.346±0.173 | 0.007 | c, b, a |
| C0P6A4 | Uncharacterized protein | 0.867±0.005 | 0.001 | 0.610±0.087 | 0.016 | 0.547±0.047 | 0.004 | a, b, b |
| C0P732 | Uncharacterized protein | 0.977±0.071 | 0.631 | 1.773±0.102 | 0.006 | 1.739±0.130 | 0.031 | b, a, a |
| C0P8F7 | Uncharacterized protein | 0.778±0.057 | 0.021 | 0.636±0.038 | 0.004 | 0.599±0.018 | 0.001 | a, b, b |
| C0P9L7 | Uncharacterized protein | 0.971±0.053 | 0.443 | 0.652±0.042 | 0.005 | 0.628±0.047 | 0.005 | a, b, b |
| C0PDC7 | Heat-shock protein 101 | 1.016±0.009 | 0.089 | 2.544±0.190 | 0.008 | 2.967±0.201 | 0.003 | b, a, a |
| C4J410 | Heat shock protein1 | 1.011±0.017 | 0.386 | 2.270±0.140 | 0.004 | 2.391±0.187 | 0.006 | b, a, a |
| C4JAJ7 | Uncharacterized protein | 1.133±0.111 | 0.173 | 0.631±0.016 | 0.001 | 0.644±0.019 | 0.001 | a, b, b |
| E1U816 | ERTC | 0.683±0.052 | 0.009 | 2.425±0.005 | 0.000 | 2.443±0.071 | 0.001 | b, a, a |
| K7TP06 | Glutamyl-tRNA reductase | 0.754±0.043 | 0.010 | 0.633±0.022 | 0.001 | 0.493±0.021 | 0.001 | a, b, c |
| K7UA57 | Uncharacterized protein | 1.001±0.100 | 0.988 | 0.479±0.055 | 0.004 | 0.448±0.042 | 0.002 | a, b, b |
| K7UHS3 | Uncharacterized protein OS=Zea mays | 1.138±0.097 | 0.132 | 2.645±0.223 | 0.010 | 5.498±0.330 | 0.002 | c, b, a |
| K7UMY0 | Uncharacterized protein | 0.763±0.055 | 0.018 | 0.631±0.020 | 0.001 | 0.616±0.027 | 0.002 | a, b, b |
| K7UWL9 | Uncharacterized protein | 0.991±0.101 | 0.891 | 0.666±0.034 | 0.003 | 0.632±0.018 | 0.001 | a, b, b |
| K7V1D4 | Uncharacterized protein | 0.678±0.037 | 0.004 | 0.555±0.028 | 0.001 | 0.575±0.022 | 0.001 | a, b, b |
| K7V2K6 | Uncharacterized protein | 1.050±0.061 | 0.291 | 1.924±0.213 | 0.017 | 2.183±0.192 | 0.009 | b, a, a |
| K7V442 | Uncharacterized protein | 0.960±0.100 | 0.560 | 0.336±0.018 | 0.000 | 0.341±0.025 | 0.000 | a, b, b |
| K7V5R0 | Uncharacterized protein | 0.933±0.029 | 0.057 | 1.631±0.087 | 0.006 | 1.940±0.026 | 0.000 | c, b, a |
| K7VJF3 | Uncharacterized protein | 0.926±0.102 | 0.334 | 1.955±0.223 | 0.018 | 2.748±0.223 | 0.005 | c, b, a |
| K7VMC6 | Uncharacterized protein | 1.008±0.100 | 0.902 | 2.062±0.187 | 0.010 | 2.155±0.212 | 0.011 | b, a, a |
| K7VQQ1 | Uncharacterized protein | 0.989±0.098 | 0.863 | 0.574±0.031 | 0.002 | 0.574±0.018 | 0.001 | a, b, b |
| K7VSC9 | 3-ketoacyl-CoA synthase | 0.834±0.024 | 0.007 | 0.570±0.028 | 0.001 | 0.487±0.022 | 0.001 | a, b, b |
| K7VZF7 | Uncharacterized protein (Fragment) | 1.246±0.098 | 0.049 | 1.731±0.176 | 0.019 | 1.667±0.027 | 0.001 | b, a, a |
| P11143 | Heat shock 70 kDa protein | 1.104±0.092 | 0.188 | 2.143±0.209 | 0.011 | 2.287±0.200 | 0.008 | b, a, a |
| Q08277 | Heat shock protein 82 | 0.948±0.099 | 0.457 | 2.019±0.140 | 0.006 | 1.832±0.150 | 0.011 | b, a, a |
| Q36284 | NADH-ubiquinone oxidoreductase chain 5 (Fragment) | 1.444±0.099 | 0.016 | 1.620±0.173 | 0.025 | 1.505±.009 | 0.000 | a, a, a |
| Q42446 | Uncharacterized protein | 1.042±0.087 | 0.492 | 0.664±0.033 | 0.003 | 0.640±0.052 | 0.007 | a, b, b |
| Q43701 | Heat shock protein 17.2 | 0.909±0.099 | 0.251 | 1.964±0.165 | 0.010 | 2.005±0.103 | 0.003 | b, a, a |
| Q5GJ59 | Terpene synthase 7 | 0.927±0.021 | 0.025 | 0.565±0.036 | 0.002 | 0.532±0.051 | 0.004 | a, b, b |
| Q6RYQ7 | Heat shock protein HSP101 | 0.940±0.022 | 0.041 | 1.711±0.195 | 0.024 | 2.034±0.190 | 0.011 | c, b, a |
| Q9FER6 | Putative legumain | 0.705±0.087 | 0.028 | 0.236±0.231 | 0.000 | 0.249±0.016 | 0.000 | a, b, b |
| Q9XE93 | Exhydrolase II | 0.983±0.053 | 0.634 | 0.558±0.030 | 0.002 | 0.535±0.031 | 0.001 | a, b, b |

*CK, control; D, drought stress; H, heat stress; DH, combined drought and heat stress.* Each value represents the average of three biological replicas. For Duncan’s Results, different characters are considered to be significant among different treatments.
