# Supplementary material for: The Difference of Physiological and Proteomic Changes in Maize Leaves Adaptation to Drought, Heat, and Combined Both Stresses
Source: Front Plant Sci. 2016 Oct 26;7:1471. doi: 10.3389/fpls.2016.01471 (PMC5080359; doi:10.3389/fpls.2016.01471)
Supplement: Supplementary file 2 [file Table2.DOC]

**Table S2︱P**roteins with significant expression level changes only under D and DH

| **Accession** | **Description** | **D/CK** | | **H/CK** | | **DH/CK** | | **Duncan's Results** |
| --- | --- | --- | --- | --- | --- | --- | --- | --- |
|  |  | **Mean (±SD)** | **P-Value** | **Mean (±SD)** | **P-Value** | **Mean (±SD)** | **P-Value** | **D, H, DH** |
| A3KLI0 | RAB17 protein | 6.235±0.906 | 0.010 | 0.932±0.087 | 0.307 | 7.756±0.606 | 0.003 | b, c, a |
| B4FTL9 | MTN3 | 0.625±0.027 | 0.002 | 0.859±0.000 | 0.001 | 0.587±0.024 | 0.001 | b, a, b |
| B4G1H1 | Uncharacterized protein | 1.859±0.140 | 0.009 | 1.061±0.104 | 0.416 | 1.824±0.154 | 0.011 | a, b, a |
| B4G1K9 | Photosystem I reaction center subunit V | 1.540±0.069 | 0.005 | 1.233±0.050 | 0.015 | 1.515±0.023 | 0.001 | a, b, a |
| B6SR64 | Uncharacterized protein | 1.787±0.104 | 0.011 | 0.869±0.065 | 0.074 | 1.702±0.000 | 0.000 | a, b, a |
| B6SRB1 | HVA22-like protein e | 1.705±0.000 | 0.000 | 0.913±0.022 | 0.020 | 1.645±0.000 | 0.000 | a, b, a |
| B6TLM5 | Glutathione S-transferase GSTU6 | 1.569±0.000 | 0.000 | 1.214±0.087 | 0.051 | 1.685±0.000 | 0.000 | b, c, a |
| B6TSV7 | Seed maturation protein | 3.323±0.347 | 0.007 | 1.059±0.098 | 0.406 | 2.150±0.153 | 0.010 | a, c, b |
| B6UAU8 | O-succinylhomoserine sulfhydrylase | 2.369±0.090 | 0.009 | 1.084±0.145 | 0.420 | 2.376±0.091 | 0.009 | a, b, a |
| B6UCP6 | Putative uncharacterized protein | 2.426±0.000 | 0.000 | 1.049±0.080 | 0.398 | 2.731±0.261 | 0.007 | a, b, a |
| B6UH30 | Uncharacterized protein | 3.135±0.000 | 0.000 | 1.125±0.020 | 0.008 | 3.067±0.115 | 0.001 | a, b, a |
| C0HI30 | Uncharacterized protein | 2.185±0.203 | 0.010 | 1.442±0.087 | 0.013 | 2.413±0.201 | 0.007 | a, b, a |
| C0PF35 | Uncharacterized protein | 5.574±0.459 | 0.003 | 1.192±0.092 | 0.068 | 5.019±0.110 | 0.000 | a, c, b |
| C4J0T9 | Uncharacterized protein | 1.992±0.000 | 0.000 | 1.249±0.046 | 0.011 | 2.315±0.109 | 0.008 | a, b, a |
| C4J477 | Dehydrin | 2.533±0.000 | 0.000 | 1.003±0.087 | 0.958 | 2.064±0.110 | 0.004 | a, c, b |
| K7TFB6 | ABA-responsive protein | 1.740±0.000 | 0.000 | 1.137±0.087 | 0.113 | 2.320±0.203 | 0.008 | b, c, a |
| K7UA28 | Uncharacterized protein | 0.634±0.055 | 0.008 | 0.692±0.021 | 0.001 | 0.658±0.015 | 0.001 | a, a, a |
| K7UIB2 | Uncharacterized protein | 0.503±0.012 | 0.000 | 0.724±0.087 | 0.032 | 0.570±0.036 | 0.002 | b, a, b |
| K7VBI0 | Uncharacterized protein | 2.135±0.153 | 0.009 | 1.285±0.087 | 0.030 | 2.074±0.111 | 0.004 | a, b, a |
| K7VNX5 | Putative cytochrome P450 superfamily protein | 1.658±0.101 | 0.008 | 1.267±0.061 | 0.017 | 2.267±0.115 | 0.003 | b, c, a |
| Q9ATM5 | Aquaporin PIP2-6 | 1.584±0.062 | 0.004 | 1.160±0.009 | 0.001 | 1.518±0.023 | 0.001 | a, b, a |

*CK, control; D, drought stress; H, heat stress; DH, combined drought and heat stress.* Each value represents the average of three biological replicas. For Duncan’s Results, different characters are considered to be significant among different treatments.
