# Supplementary material for: The Difference of Physiological and Proteomic Changes in Maize Leaves Adaptation to Drought, Heat, and Combined Both Stresses
Source: Front Plant Sci. 2016 Oct 26;7:1471. doi: 10.3389/fpls.2016.01471 (PMC5080359; doi:10.3389/fpls.2016.01471)
Supplement: Supplementary file 3 [file Table3.DOC]

**Table S3︱P**roteins with significant expression level changes only under D and H

|  |  |  |  |  |  |  |  |  |
| --- | --- | --- | --- | --- | --- | --- | --- | --- |
| Accession | Description | D/CK | | H/CK | | DH/CK | | Duncan's Results |
|  |  | Mean (±SD) | P-Value | Mean (±SD) | P-Value | Mean (±SD) | P-Value | D, H, DH |
| C0HE41 | Uncharacterized protein | 2.103±0.178 | 0.009 | 1.527±0.047 | 0.003 | 0.922±0.092 | 0.278 | a, b, c |

*CK, control; D, drought stress; H, heat stress; DH, combined drought and heat stress.* Each value represents the average of three biological replicas. For Duncan’s Results, different characters are considered to be significant among different treatments.
