# Supplementary material for: The Difference of Physiological and Proteomic Changes in Maize Leaves Adaptation to Drought, Heat, and Combined Both Stresses
Source: Front Plant Sci. 2016 Oct 26;7:1471. doi: 10.3389/fpls.2016.01471 (PMC5080359; doi:10.3389/fpls.2016.01471)
Supplement: Supplementary file 4 [file Table4.DOC]

**Table S4︱P**roteins with significant expression level changes only under H

| Accession | Description | D/CK | | H/CK | | DH/CK | | Duncan's Results |
| --- | --- | --- | --- | --- | --- | --- | --- | --- |
|  |  | Mean (±SD) | P-Value | Mean (±SD) | P-Value | Mean (±SD) | P-Value | D,H,DH |
| B4FP70 | Small nuclear ribonucleoprotein-associated protein | 1.099±0.166 | 0.099 | 1.597±0.103 | 0.009 | 1.194±0.087 | 0.061 | b, a, b |
| B6SJF9 | Cortical cell-delineating protein | 0.829±0.087 | 0.076 | 0.641±0.021 | 0.001 | 0.749±0.018 | 0.002 | a, b, a |
| B6SQ41 | Putative uncharacterized protein | 1.105±0.173 | 0.404 | 0.651±0.026 | 0.002 | 0.695±0.020 | 0.001 | a, b, b |
| B6SSK4 | Uncharacterized protein | 0.992±0.089 | 0.890 | 1.696±0.087 | 0.005 | 1.411±0.018 | 0.001 | c, a, b |
| B6SSU0 | NADP-dependent oxidoreductase P2 | 0.949±0.080 | 0.383 | 0.646±0.044 | 0.005 | 0.703±0.004 | 0.000 | a, b, b |
| B6TPW4 | Eukaryotic translation initiation factor 2 beta subunit | 1.091±0.089 | 0.218 | 1.540±0.052 | 0.003 | 1.399±0.024 | 0.001 | c, a, b |
| B6U0H4 | SET domain containing protein | 1.040±0.087 | 0.510 | 0.623±0.023 | 0.001 | 0.685±0.026 | 0.002 | a, b, b |
| B6U2X6 | Mitochondrial import receptor subunit TOM22 | 1.308±0.097 | 0.031 | 1.523±0.023 | 0.001 | 1.479±0.076 | 0.008 | b, a, a |
| B6U4W2 | Putative uncharacterized protein | 0.805±0.009 | 0.001 | 0.636±0.032 | 0.003 | 0.717±0.029 | 0.003 | a, c, b |
| C0P8S3 | Uncharacterized protein | 0.992±0.092 | 0.894 | 0.659±0.011 | 0.000 | 0.675±0.018 | 0.001 | a, b, b |
| C4J3B1 | Pectinesterase | 1.026±0.039 | 0.368 | 0.657±0.006 | 0.000 | 0.728±0.046 | 0.009 | a, c, b |
| K7UVS5 | Uncharacterized protein | 0.960±0.008 | 0.012 | 0.639±0.015 | 0.001 | 0.711±0.017 | 0.001 | a, c, b |
| K7VC62 | Iron-sulfur assembly protein IscA, mRNA | 0.962±0.104 | 0.591 | 0.616±0.026 | 0.002 | 0.667±0.007 | 0.000 | a, b, b |
| K7VG67 | Uncharacterized protein | 0.916±0.087 | 0.237 | 0.611±0.017 | 0.001 | 0.742±0.030 | 0.004 | a, c, b |
| Q1KK93 | NADH-ubiquinone oxidoreductase chain 4 | 1.289±0.139 | 0.069 | 1.684±0.114 | 0.009 | 1.476±0.023 | 0.001 | b, a, a |

*CK, control; D, drought stress; H, heat stress; DH, combined drought and heat stress.* Each value represents the average of three biological replicas. For Duncan’s Results, different characters are considered to be significant among different treatments.
