# Supplementary material for: The Difference of Physiological and Proteomic Changes in Maize Leaves Adaptation to Drought, Heat, and Combined Both Stresses
Source: Front Plant Sci. 2016 Oct 26;7:1471. doi: 10.3389/fpls.2016.01471 (PMC5080359; doi:10.3389/fpls.2016.01471)
Supplement: Supplementary file 5 [file Table5.DOC]

**Table S5︱The P**roteins with significant expression level changes only under D

| Accession | Description | D/CK | | H/CK | | DH/CK | | Duncan's Results |
| --- | --- | --- | --- | --- | --- | --- | --- | --- |
|  |  | Mean (±SD) | P-Value | Mean (±SD) | P-Value | Mean (±SD) | P-Value | D, H, DH |
| B4F9F7 | Uncharacterized protein | 1.540±0.061 | 0.004 | 1.221±0.132 | 0.102 | 1.221±0.105 | 0.068 | a, b, b |
| B4F9W3 | Uncharacterized protein | 1.557±0.000 | 0.000 | 1.020±0.100 | 0.763 | 1.052±0.000 | 0.423 | a, b, b |
| B4FSE1 | Thiamine thiazole synthase, chloroplastic | 0.658±0.036 | 0.004 | 0.826±0.079 | 0.063 | 0.727±0.066 | 0.019 | b, a, b |
| B4FT03 | Uncharacterized protein | 1.687±0.036 | 0.001 | 0.934±0.036 | 0.087 | 1.269±0.036 | 0.006 | a, c, b |
| B4FU26 | Uncharacterized protein | 3.397±0.036 | 0.000 | 0.855±0.036 | 0.020 | 1.056±0.036 | 0.115 | a, c, b |
| B4G206 | Uncharacterized protein | 1.532±0.026 | 0.001 | 0.998±0.036 | 0.932 | 1.145±0.026 | 0.011 | a, c, b |
| B6SKV1 | Delta 1-pyrroline-5-carboxylate synthetase | 2.396±0.036 | 0.000 | 1.157±0.036 | 0.017 | 1.382±0.036 | 0.003 | a, c, b |
| B6SRV6 | Stachyose synthase | 1.593±0.036 | 0.001 | 0.903±0.036 | 0.043 | 1.387±0.036 | 0.003 | a, c, b |
| B6SU65 | 3-oxo-5-alpha-steroid 4-dehydrogenase 2 | 1.542±0.036 | 0.001 | 0.906±0.079 | 0.177 | 1.023±0.036 | 0.384 | a, c, b |
| B6SUK1 | Ligatin OS=Zea mays | 0.626±0.036 | 0.003 | 0.727±0.036 | 0.006 | 0.751±0.036 | 0.007 | b, a, a |
| B6SYY2 | Stachyose synthase | 3.095±0.041 | 0.000 | 0.878±0.066 | 0.084 | 1.282±0.036 | 0.005 | a, c, b |
| B6SZN0 | MtN19-like protein | 2.691±0.036 | 0.000 | 0.889±0.036 | 0.033 | 1.229±0.036 | 0.008 | a, c, b |
| B6T9X8 | Protein phosphatase 2C | 2.148±0.036 | 0.000 | 1.372±0.066 | 0.010 | 1.460±0.036 | 0.002 | a, b, b |
| B6TCX6 | Putative uncharacterized protein | 1.701±0.036 | 0.001 | 0.960±0.036 | 0.195 | 1.015±0.036 | 0.546 | a, b, b |
| B6TRW8 | Dihydrolipoyllysine-residue succinyltransferase component of 2-oxoglutarate dehydrogenase complex | 0.665±0.036 | 0.004 | 1.199±0.079 | 0.049 | 1.364±0.036 | 0.003 | c, b, a |
| B6TU95 | Putative uncharacterized protein | 1.802±0.139 | 0.010 | 1.036±0.036 | 0.226 | 1.450±0.033 | 0.002 | a, c, b |
| C0P496 | Uncharacterized protein | 0.661±0.036 | 0.004 | 0.923±0.036 | 0.066 | 0.687±0.036 | 0.004 | b, a, b |
| C0P4N4 | Uncharacterized protein | 1.681±0.066 | 0.003 | 1.185±0.036 | 0.012 | 1.336±0.036 | 0.004 | a, c, b |
| C0P6X7 | Uncharacterized protein | 1.925±0.036 | 0.001 | 0.674±0.036 | 0.004 | 0.893±0.036 | 0.036 | a, c, b |
| C0P8H1 | Uncharacterized protein | 2.064±0.036 | 0.000 | 0.971±0.036 | 0.298 | 1.096±0.036 | 0.044 | a, c, b |
| C0PBJ7 | Uncharacterized protein | 1.755±0.036 | 0.001 | 1.042±0.036 | 0.181 | 1.411±0.036 | 0.003 | a, c, b |
| C0PLS3 | Uncharacterized protein | 1.650±0.017 | 0.000 | 1.199±00.036 | 0.011 | 0.967±0.036 | 0.254 | a, b, c |
| C4J9Y2 | Uncharacterized protein | 0.659±0.010 | 0.000 | 1.015±0.036 | 0.546 | 0.701±0.036 | 0.005 | b, a, b |
| K7U2U2 | Uncharacterized protein | 1.707±0.036 | 0.001 | 0.980±0.036 | 0.438 | 0.928±0.036 | 0.074 | a, b, b |
| K7UT58 | Putative glycogen synthase kinase family protein | 0.542±0.036 | 0.002 | 0.669±0.036 | 0.004 | 0.783±0.036 | 0.009 | c, b, a |
| K7VB23 | Uncharacterized protein | 2.723±0.139 | 0.002 | 1.078±0.036 | 0.064 | 1.046±0.036 | 0.158 | a, b, b |
| P60138 | Photosystem II reaction center protein L | 1.642±0.036 | 0.001 | 0.844±0.036 | 0.017 | 0.926±0.036 | 0.071 | a, c, b |
| P69523 | Cytochrome b559 subunit beta | 1.706±0.096 | 0.006 | 0.990±0.036 | 0.678 | 1.027±0.036 | 0.324 | a, b, b |

*CK, control; D, drought stress; H, heat stress; DH, combined drought and heat stress.* Each value represents the average of three biological replicas. For Duncan’s Results, different characters are considered to be significant among different treatments.
