# Supplementary material for: The Difference of Physiological and Proteomic Changes in Maize Leaves Adaptation to Drought, Heat, and Combined Both Stresses
Source: Front Plant Sci. 2016 Oct 26;7:1471. doi: 10.3389/fpls.2016.01471 (PMC5080359; doi:10.3389/fpls.2016.01471)
Supplement: Supplementary file 6 [file Table6.DOC]

**Table S6︱The P**roteins with significant expression level changes only under DH

| **Accession** | **Description** | **D/CK** | | **H/CK** | | **DH/CK** | | **Duncan's Results** |
| --- | --- | --- | --- | --- | --- | --- | --- | --- |
|  |  | Mean (±SD) | **P-Value** | Mean (±SD) | **P-Value** | Mean (±SD) | **P-Value** | D, H, DH |
| A6YSM3 | PL3K2 | 1.309±0.038 | 0.005 | 1.437±0.020 | 0.001 | 2.375±0.063 | 0.001 | c, b, a |
| B4F8Z1 | Uncharacterized protein | 0.741±0.068 | 0.022 | 0.700±0.020 | 0.001 | 0.633±0.045 | 0.005 | a, ab, b |
| B4F988 | FtsH6-Zea mays FtsH protease | 1.090±0.083 | 0.203 | 1.423±0.082 | 0.012 | 3.574±0.270 | 0.004 | b, a, a |
| B4FAB3 | GTP-binding protein | 0.833±0.068 | 0.051 | 0.697±0.020 | 0.001 | 0.646±0.045 | 0.005 | a, b, b |
| B4FHM6 | Uncharacterized protein | 0.786±0.038 | 0.010 | 0.802±0.084 | 0.055 | 0.606±0.046 | 0.004 | a, a, b |
| B4FKD7 | Uncharacterized protein | 1.411±0.038 | 0.003 | 1.241±0.020 | 0.002 | 1.508±0.029 | 0.001 | b, c, a |
| B4FKG5 | Abscisic stress ripening protein 2 | 1.435±0.038 | 0.003 | 1.289±0.020 | 0.002 | 1.621±0.063 | 0.003 | b, c, a |
| B4FMW6 | Uncharacterized protein | 0.851±0.038 | 0.021 | 0.736±0.084 | 0.032 | 0.639±0.045 | 0.005 | a, ab, b |
| B4FP20 | Uncharacterized protein | 0.955±0.038 | 0.178 | 0.767±0.020 | 0.002 | 0.654±0.063 | 0.011 | a, b, c |
| B4FRG9 | Uncharacterized protein | 0.814±0.038 | 0.014 | 0.689±0.020 | 0.001 | 0.642±0.063 | 0.010 | a, b, b |
| B4FUH2 | Aspartate aminotransferase | 1.336±0.038 | 0.004 | 1.096±0.020 | 0.014 | 1.524±0.063 | 0.005 | b, c, a |
| B4FZN7 | Uncharacterized protein | 0.682±0.038 | 0.005 | 0.688±0.020 | 0.001 | 0.605±0.063 | 0.009 | a, a, a |
| B4G0P6 | Uncharacterized protein | 0.916±0.038 | 0.062 | 0.689±0.020 | 0.001 | 0.656±0.045 | 0.006 | a, b, b |
| B6ETR5 | Asparagine synthetase | 1.461±0.068 | 0.007 | 1.274±0.084 | 0.030 | 2.363±0.021 | 0.010 | b, b, a |
| B6SP03 | Putative uncharacterized protein | 0.993±0.038 | 0.781 | 1.407±0.020 | 0.001 | 1.748±0.063 | 0.002 | c, b, a |
| B6SR73 | Tubulin alpha-6 chain | 1.138±0.038 | 0.025 | 1.280±0.020 | 0.002 | 1.680±0.063 | 0.003 | c, b, a |
| B6SSB7 | Protein kinase | 1.150±0.038 | 0.021 | 1.385±0.020 | 0.001 | 1.622±0.063 | 0.003 | c, b, a |
| B6ST41 | RPT2-like protein | 0.677±0.038 | 0.005 | 0.837±0.020 | 0.005 | 0.632±0.063 | 0.010 | b, a, b |
| B6T3Q3 | Ethylene-responsive protein | 1.205±0.038 | 0.011 | 1.430±0.084 | 0.013 | 1.984±0.063 | 0.001 | c, b, a |
| B6T8R8 | Phosphoethanolamine N-methyltransferase | 1.249±0.038 | 0.008 | 1.326±0.082 | 0.021 | 1.728±0.063 | 0.003 | b, b, a |
| B6TA17 | Kinesin heavy chain isolog | 1.005±0.038 | 0.842 | 0.709±0.020 | 0.002 | 0.647±0.045 | 0.005 | a, b, b |
| B6TB13 | Indole-3-acetate beta-glucosyltransferase | 0.860±0.038 | 0.024 | 0.704±0.084 | 0.026 | 0.652±0.045 | 0.006 | a, b, b |
| B6TC25 | Gibberellin receptor GID1L2 | 0.957±0.038 | 0.190 | 0.734±0.020 | 0.002 | 0.66±0.063 | 0.011 | a, b, b |
| B6TGE4 | Putative uncharacterized protein | 1.134±0.038 | 0.026 | 1.429±0.084 | 0.013 | 1.775±0.063 | 0.002 | c, b, a |
| B6TGK8 | Uncharacterized protein | 1.085±0.038 | 0.061 | 0.688±0.020 | 0.001 | 0.644±0.045 | 0.005 | a, b, b |
| B6TGW6 | Putative uncharacterized protein | 1.078±0.038 | 0.071 | 1.421±0.084 | 0.013 | 1.552±0.045 | 0.002 | c, b, a |
| B6TIK3 | Stress protein | 1.249±0.068 | 0.024 | 1.470±0.084 | 0.011 | 1.760±0.063 | 0.002 | c, b, a |
| B6TJM1 | 3-isopropylmalate dehydrogenase | 1.275±0.038 | 0.006 | 1.363±0.084 | 0.017 | 1.501±0.063 | 0.005 | b, b, a |
| B6TK50 | Armadillo/beta-catenin-like repeat family protein | 0.950±0.038 | 0.151 | 1.430±0.020 | 0.001 | 1.513±0.063 | 0.005 | b, b, a |
| B6TWG6 | COP9 signalosome complex subunit 6a | 0.943±0.038 | 0.123 | 1.392±0.020 | 0.001 | 1.751±0.043 | 0.001 | c, b, a |
| B6TXS5 | Photosystem I reaction center subunit N | 1.358±0.068 | 0.012 | 1.311±0.084 | 0.024 | 1.737±0.063 | 0.002 | b, b, a |
| B6U1W0 | CBS domain containing protein | 1.385±0.038 | 0.003 | 1.401±0.020 | 0.001 | 1.621±0.036 | 0.001 | b, b, a |
| B6UBQ9 | Ankyrin protein kinase-like | 0.774±0.068 | 0.029 | 0.803±0.084 | 0.056 | 0.648±0.063 | 0.011 | ab, a b |
| B6UBW7 | Stachyose synthase | 0.988±0.038 | 0.641 | 1.404±0.084 | 0.014 | 1.541±0.045 | 0.002 | c, b, a |
| B8A161 | Nucleosome/chromatin assembly factor group A | 1.132±0.038 | 0.027 | 1.287±0.084 | 0.028 | 1.554±0.050 | 0.003 | c, b, a |
| C0HE53 | Uncharacterized protein | 0.728±0.071 | 0.022 | 0.767±0.020 | 0.002 | 0.638±0.045 | 0.005 | ab, a, b |
| C0HG57 | Uncharacterized protein | 0.753±0.038 | 0.008 | 0.700±0084 | 0.025 | 0.624±0.045 | 0.005 | a, ab, b |
| C0PD01 | Uncharacterized protein | 0.760±0.068 | 0.026 | 0.702±0.020 | 0.001 | 0.665±0.050 | 0.007 | a, a, a |
| C0PFV7 | Uncharacterized protein | 0.841±0.038 | 0.019 | 0.708±0.084 | 0.027 | 0.581±0.063 | 0.008 | b, b, a |
| C0PNI2 | Uncharacterized protein | 0.707±0.038 | 0.006 | 0.770±0.020 | 0.003 | 0.665±0.040 | 0.005 | ab, a, b |
| C4JBB8 | Uncharacterized protein | 1.192±0.038 | 0.013 | 1.426±0.103 | 0.019 | 1.531±0.063 | 0.005 | b, a, a |
| D1MN58 | ABA-, stress-and fruit-ripening inducible-like protein | 1.479±0.038 | 0.002 | 1.231±0.084 | 0.042 | 1.617±0.063 | 0.004 | b, c, a |
| F1DJV0 | BZIP transcription factor (Fragment) | 1.169±0.038 | 0.017 | 1.409±0.084 | 0.014 | 1.768±0.063 | 0.002 | c, b, a |
| K7TQ71 | Uncharacterized protein | 0.865±0.049 | 0.041 | 0.719±0.020 | 0.002 | 0.606±0.063 | 0.009 | a, b, c |
| K7U346 | Uncharacterized protein | 1.047±0.038 | 0.166 | 1.459±0.084 | 0.011 | 1.654±0.045 | 0.002 | c, b, a |
| K7U4Y5 | Uncharacterized protein | 1.182±0.068 | 0.044 | 1.339±0.084 | 0.020 | 1.845±0.142 | 0.009 | b, b, a |
| K7UL40 | Uncharacterized protein | 0.817±0.038 | 0.014 | 0.736±0.020 | 0.002 | 0.657±0.063 | 0.011 | a, ab, b |
| K7USR3 | Uncharacterized protein | 0.882±0.038 | 0.033 | 0.789±0.020 | 0.003 | 0.657±0.038 | 0.004 | a, b, c |
| K7UWZ6 | Uncharacterized protein | 0.753±0.038 | 0.008 | 0.851±0.020 | 0.006 | 0.664±0.033 | 0.003 | b, a, c |
| K7UZF0 | Uncharacterized protein | 1.426±0.071 | 0.009 | 1.455±0.020 | 0.001 | 1.502±0.045 | 0.003 | a, a, a |
| K7VF90 | Uncharacterized protein | 0.995±0.038 | 0.842 | 0.698±0.084 | 0.025 | 0.57±0.063 | 0.007 | a, b, b |
| K7VQU8 | Putative DEAD-box ATP-dependent RNA helicase family protein | 0.954±0.038 | 0.172 | 0.727±0.020 | 0.002 | 0.623±0.045 | 0.005 | a, b, c |
| K7VT58 | Fasciclin-like arabinogalactan protein 7 | 0.983±0.038 | 0.521 | 0.737±0.020 | 0.002 | 0.614±0.063 | 0.009 | a, b, c |
| O24595 | Glutathione transferase | 0.875±0.068 | 0.087 | 1.429±0.084 | 0.013 | 1.583±0.063 | 0.004 | c, b, a |
| P17571 | Nitrate reductase [NADH] (Fragment) | 0.717±0.038 | 0.006 | 0.713±0.020 | 0.002 | 0.567±0.063 | 0.007 | a, a, b |
| P24067 | Luminal-binding protein 2 | 1.022±0.038 | 0.423 | 1.381±0.084 | 0.016 | 1.560±0.063 | 0.004 | c, b, a |
| P93518 | PRm 3 | 1.484±0.068 | 0.007 | 1.152±0.084 | 0.089 | 1.752±0.063 | 0.002 | b, c, a |
| Q5D1L6 | KZM2 | 1.371±0.038 | 0.004 | 1.479±0.020 | 0.001 | 1.631±0.063 | 0.003 | b, c, a |
| Q9FPK7 | Inositol-3-phosphate synthase | 1.256±0.068 | 0.023 | 1.313±0.020 | 0.001 | 1.674±0.059 | 0.003 | b, b, a |

*CK, control; D, drought stress; H, heat stress; DH, combined drought and heat stress.* Each value represents the average of three biological replicas. For Duncan’s Results, different characters are considered to be significant among different treatments.
