# Supplementary material for: The Difference of Physiological and Proteomic Changes in Maize Leaves Adaptation to Drought, Heat, and Combined Both Stresses
Source: Front Plant Sci. 2016 Oct 26;7:1471. doi: 10.3389/fpls.2016.01471 (PMC5080359; doi:10.3389/fpls.2016.01471)
Supplement: Supplementary file 7 [file Table7.DOC]

**Table S7︱Maize proteins corresponding to rice proteins in network of protein interaction under D stress.**

| Maize query sequence | Rice query sequence | STRING protein | Identity | Bitscore |
| --- | --- | --- | --- | --- |
| P60138 | 3131418 | photosystem II protein L | 100% | 77 |
| P69523 | 3131414 | cytochrome b559 beta chain | 100% | 81.3 |
| Q42376 | 4326129 | late embryogenesis abundant protein, group 3 | 58% | 180 |
| Q9ATM5 | 4343119 | aquaporin protein | 91% | 533 |
| A3KLI0 | 4350453 | dehydrin | 61% | 102 |
| B4F7X5 | 4341121 | reticulon domain containing protein | 77% | 382 |
| B4F9F7 | 4330194 | serine esterase family protein | 79% | 381 |
| B4F9W3 | 4343709 | chlorophyll A-B binding protein | 97% | 311 |
| B4FSE1 | 4343443 | FAD dependent oxidoreductase domain containing protein | 89% | 558 |
| B4FT03 | 4336460 | expressed protein | 87% | 737 |
| B4FT63 | 4350255 | uncharacterized protein ycf53 | 68% | 358 |
| B4FTL9 | 4352201 | nodulin MtN3 family protein, putative | 78% | 409 |
| B4FU26 | 4332401 | haloacid dehalogenase-like hydrolase family protein | 89% | 428 |
| B4G1H1 | 4330265 | dehydrin | 57% | 190 |
| B4G1K9 | 4347395 | photosystem I reaction center subunit | 86% | 222 |
| B4G206 | 4347696 | ki1 protein | 85% | 577 |
| B5U8J8 | 4332506 | asparagine synthetase | 84% | 1055 |
| B6SID7 | 4326129 | late embryogenesis abundant protein, group 3 | 59% | 174 |
| B6SKV1 | 4324853 | amino acid kinase | 88% | 1307 |
| B6SMU2 | 4341482 | expressed protein | 81% | 114 |
| B6SQF4 | OsI_34149 | alpha-galactosidase precursor | 84% | 681 |
| B6SRB1 | 4350556 | HVA22 | 80% | 231 |
| B6SRV6 | 4342703 | uncharacterized glycosyltransferase | 82% | 1246 |
| B6SU65 | 4342458 | 3-oxo-5-alpha-steroid 4-dehydrogenase | 69% | 313 |
| B6SUK1 | B1012D10.2 | translation machinery-associated protein 20 | 97% | 340 |
| B6SWZ1 | 4343590 | transporter family protein | 94% | 930 |
| B6SYY2 | RFS | uncharacterized glycosyltransferase | 82% | 1331 |
| B6SZN0 | 4346175 | mtN19 | 62% | 520 |
| B6T531 | 4332957 | RNA recognition motif containing protein | 73% | 350 |
| B6T9X8 | 4327465 | protein phosphatase 2C | 74% | 540 |
| B6TCX6 | 4337743 | expressed protein | 54% | 127 |
| B6TEH8 | 4339389 | anthocyanidin 5,3-O-glucosyltransferase | 69% | 615 |
| B6TLM5 | 4325643 | glutathione S-transferase | 71% | 319 |
| B6TRW8 | 4335689 | dihydrolipoyllysine-residue succinyltransferase component of 2-oxoglutarate dehydrogenase complex | 87% | 785 |
| B6TSV7 | 4345287 | late embryogenesis abundant group 1 | 71% | 187 |
| B6TU95 | 4331521 | expressed protein | 80% | 181 |
| B6U471 | 4344001 | RNA recognition motif containing protein | 72% | 351 |
| B6UAN2 | OsJ_00883 | powdery mildew resistant protein 5 | 74% | 544 |
| B6UAU8 | 4349123 | cystathionine gamma-synthase | 89% | 774 |
| B6UCG5 | 4326610 | psbP-related thylakoid lumenal protein 4 | 89% | 281 |
| B6UH30 | 4339065 | phosphatidylethanolamine-binding protein | 78% | 262 |
| C0HE41 | 4340424 | flavin-containing monooxygenase family protein | 63% | 622 |
| C0HI30 | 4339812 | NAD dependent epimerase/dehydratase family protein | 89% | 653 |
| C0P496 | OsI_00939 | expressed protein | 83% | 356 |
| C0P4N4 | RFS | uncharacterized glycosyltransferase | 82% | 1337 |
| C0P6X7 | 4347225 | 1-aminocyclopropane-1-carboxylate oxidase protein | 87% | 514 |
| C0P8H1 | 4328018 | glycosyl hydrolases, putative, expressed | 87% | 245 |
| C0PBJ7 | 4339675 | CTP synthase | 89% | 1097 |
| C0PLS3 | OsJ_02493 | chlorophyll A-B binding protein | 97% | 312 |
| C0PN61 | 4347611 | glycosyl hydrolase family 29 | 82% | 458 |
| C4J0T9 | OsJ_04937 | haloacid dehalogenase-like hydrolase family protein | 83% | 411 |
| C4J477 | 4330265 | dehydrin | 56% | 181 |
| C4J9Y2 | 4349769 | stem-specific protein TSJT1 | 89% | 437 |
| K7TFB6 | 4352207 | GRAM domain containing protein | 85% | 422 |
| K7U2U2 | 4346288 | AAA-type ATPase family protein | 76% | 1675 |
| K7U7W9 | 4332690 | magnesium-chelatase | 93% | 2608 |
| K7UFK0 | 4329593 | decarboxylase | 63% | 136 |
| K7UT58 | 4327108 | CGMC_GSK.1 - CGMC includes CDA, MAPK, GSK3, and CLKC kinases | 96% | 772 |
| K7VB23 | 4333230 | mitotic checkpoint protein | 95% | 497 |
| K7VBI0 | 4326743 | AAA-type ATPase family protein | 85% | 1382 |
| K7VNX5 | 4327607 | cytochrome P450 | 83% | 836 |
| O24626 | 4332500 | omega-3 fatty acid desaturase, chloroplast precursor | 84% | 671 |
