# Supplementary material for: The Difference of Physiological and Proteomic Changes in Maize Leaves Adaptation to Drought, Heat, and Combined Both Stresses
Source: Front Plant Sci. 2016 Oct 26;7:1471. doi: 10.3389/fpls.2016.01471 (PMC5080359; doi:10.3389/fpls.2016.01471)
Supplement: Supplementary file 8 [file Table8.DOC]

**Table S8︱Maize proteins corresponding to rice proteins in network of protein interaction under heat.**

| Maize query sequence | Rice query  sequence | STRING protein | Identity | Bitscore |
| --- | --- | --- | --- | --- |
| K7UHS3 | OsI_21690 | retrotransposon protein | 35% | 236 |
| B6T630 | 4340361 | expressed protein | 42% | 288 |
| B6TKI8 | 4342676 | expressed protein | 48% | 142 |
| B4FUV7 | 4328767 | expressed protein | 50% | 131 |
| Q42376 | 4326129 | late embryogenesis abundant protein, group 3 | 58% | 180 |
| B6SSH9 | 4347709 | ribonuclease T2 family domain containing protein | 58% | 271 |
| Q5GJ59 | TPS13 | terpene synthase | 60% | 637 |
| B6SZ50 | OsI_19337 | retrotransposon protein, putative, LINE subclass | 61% | 238 |
| C0HE41 | 4340424 | flavin-containing monooxygenase family protein | 63% | 622 |
| K7UFK0 | 4329593 | decarboxylase | 63% | 136 |
| B4FT63 | 4350255 | uncharacterized protein ycf53 | 68% | 358 |
| C0P8S3 | 4349027 | expressed protein | 68% | 227 |
| B6TEH8 | 4339389 | anthocyanidin 5,3-O-glucosyltransferase | 69% | 615 |
| B6SJF9 | 4336616 | LTPL122 - Protease inhibitor/seed storage/LTP family protein precursor | 70% | 158 |
| B6T3D8 | 4338780 | monooxygenase | 72% | 461 |
| B6U471 | 4344001 | RNA recognition motif containing protein | 72% | 351 |
| B6TIP9 | HSP18.0 | hsp20/alpha crystallin family protein | 72% | 196 |
| B6TQD6 | 4340661 | hsp20/alpha crystallin family protein | 72% | 165 |
| B4FZD7 | 4333513 | single-stranded DNA-binding protein | 73% | 434 |
| B6T531 | 4332957 | RNA recognition motif containing protein | 73% | 350 |
| B6TYT3 | OsI_13535 | cysteine protease 1 precursor | 73% | 459 |
| B6SJR4 | 4341426 | OsFBK16 - F-box domain and kelch repeat containing protein | 73% | 536 |
| B6UAN2 | OsJ_00883 | powdery mildew resistant protein 5 | 74% | 544 |
| B4F9E8 | 4330933 | hsp20/alpha crystallin family protein | 74% | 233 |
| B6SQN7 | OsI_27179 | expressed protein | 74% | 340 |
| B6UHH1 | 4349779 | expressed protein | 74% | 213 |
| B6SZA8 | 4329382 | expressed protein | 75% | 364 |
| B4FDE5 | 4335011 | CBS domain containing membrane protein | 76% | 176 |
| B6SV61 | 4349565 | inactive receptor kinase At2g26730 precursor | 76% | 1106 |
| B6TMQ1 | 4345863 | RNA recognition motif containing protein | 76% | 258 |
| B4FQS7 | 4330786 | heat shock 22 kDa protein, mitochondrial precursor | 76% | 305 |
| B6T649 | 4330786 | heat shock 22 kDa protein, mitochondrial precursor | 76% | 298 |
| B4F7X5 | 4341121 | reticulon domain containing protein | 77% | 382 |
| B6TQG2 | 4344249 | stress-related protein | 77% | 340 |
| Q42446 | 4341942 | glycosyl hydrolases family 16 | 77% | 445 |
| B4FGY0 | 4325302 | SGS domain containing protein | 77% | 346 |
| B7ZEQ0 | 4332237 | hsp20/alpha crystallin family protein | 77% | 336 |
| B6UHB6 | 4334439 | retrotransposon protein, putative, Ty3-gypsy subclass | 78% | 128 |
| B4F9K4 | HSP18.0 | hsp20/alpha crystallin family protein | 78% | 220 |
| B4FLE3 | 4332979 | CS domain containing protein | 78% | 242 |
| B6SSU0 | OsI_23614 | NADP-dependent oxidoreductase | 79% | 561 |
| B6TMB1 | 4325373 | proteins of unknown function domain containing protein | 79% | 629 |
| B6U2Y8 | 4334316 | ribose-5-phosphate isomerase A | 79% | 385 |
| B6UDP0 | 4327971 | OsPDIL1-4 protein disulfide isomerase PDIL1-4 | 79% | 826 |
| C0P8F7 | 4334343 | mTERF domain containing protein | 79% | 487 |
| K7UVS5 | 4329591 | U-box domain containing protein | 79% | 605 |
| B6TIJ3 | 4331115 | thioredoxin family protein | 79% | 405 |
| B6U100 | 4329388 | peptidyl-prolyl isomerase | 79% | 962 |
| K7VZF7 | 4332853 | SNF2 family N-terminal domain containing protein | 79% | 2011 |
| B4FPQ2 | 4347851 | dihydroneopterin aldolase | 80% | 212 |
| B6SQ41 | LOC_Os01g60830.1 | expressed protein | 80% | 279 |
| B6T026 | 4330227 | LTPL113 - Protease inhibitor/seed storage/LTP family protein precursor | 80% | 177 |
| C0P5X6 | 4351224 | AAA-type ATPase family protein | 80% | 690 |
| B4FI16 | 4325310 | phosphatidate cytidylyltransferase | 81% | 710 |
| B6SMU2 | 4341482 | expressed protein | 81% | 114 |
| B6UE38 | 4342233 | Purple acid phosphatase | 81% | 1073 |
| B4F976 | 4332363 | hsp20/alpha crystallin family protein | 81% | 246 |
| B6T2J9 | 4325697 | hsp20/alpha crystallin family protein | 81% | 232 |
| B6TA56 | 4340005 | histone H1 | 81% | 143 |
| B6TLK8 | 4332363 | hsp20/alpha crystallin family protein | 81% | 249 |
| C0P732 | 4330134 | heat shock protein STI | 81% | 921 |
| B4FKX6 | 4347912 | oxidoreductase, short chain dehydrogenase/reductase family domain containing family | 82% | 540 |
| B6TY06 | 4352868 | RNA recognition motif containing protein | 82% | 149 |
| B8Y6I0 | 4338274 | pentatricopeptide | 82% | 1311 |
| C0PN61 | 4347611 | glycosyl hydrolase family 29 | 82% | 458 |
| K7V442 | OsJ_02899 | phosphoglycerate mutase | 82% | 205 |
| K7VSC9 | OsJ_02069 | 3-ketoacyl-CoA synthase | 82% | 774 |
| B4G250 | 4332363 | hsp20/alpha crystallin family protein | 82% | 253 |
| B6TTV8 | 4339664 | ubiquitin carboxyl-terminal hydrolase, family 1 | 83% | 745 |
| C4JAJ7 | 4347545 | Os9bglu31 - beta-glucosidase, dhurrinase, similar to G. max hydroxyisourate hydrolase | 83% | 866 |
| E1U816 | 4341420 | endothelial differentiation-related factor 1 | 83% | 212 |
| B6SQF4 | OsI_34149 | alpha-galactosidase precursor | 84% | 681 |
| B6U0H4 | 4330640 | SET domain-containing protein | 84% | 763 |
| B6U4W2 | OsI_32127 | expressed protein | 84% | 395 |
| O24626 | 4332500 | omega-3 fatty acid desaturase | 84% | 671 |
| B5U8J8 | 4332506 | asparagine synthetase | 84% | 1055 |
| B6SIA6 | 4342247 | expressed protein | 84% | 141 |
| B6UET0 | 4333985 | peptidase, M50 family | 84% | 924 |
| B4FA43 | 4330554 | aspartic proteinase nepenthesin-1 precursor | 85% | 253 |
| Q9FER6 | OsJ_02232 | vacuolar-processing enzyme precursor | 85% | 838 |
| B6THJ5 | 4341866 | phosphosulfolactate synthase-related protein | 85% | 506 |
| B4F8F5 | 4330523 | fatty acid desaturase | 86% | 718 |
| B6U2X6 | 4329408 | expressed protein | 86% | 120 |
| C0P9L7 | 4341497 | copine-1 | 86% | 448 |
| K7V5R0 | 4326516 | hexokinase | 86% | 608 |
| B4FFS8 | 4352868 | RNA recognition motif containing protein | 87% | 155 |
| B6STA5 | 4352868 | RNA recognition motif containing protein | 87% | 160 |
| C4J3B1 | 4324842 | pectinesterase | 87% | 466 |
| Q9XE93 | 4334116 | periplasmic beta-glucosidase precursor | 87% | 1118 |
| B6TTC8 | 4325697 | hsp20/alpha crystallin family protein | 87% | 249 |
| B4G1V3 | 4344001 | RNA recognition motif containing protein | 88% | 284 |
| B8A0I4 | 4350988 | GTP-binding protein | 88% | 628 |
| K7VG67 | 4341733 | OsGrx_C4 - glutaredoxin subgroup I | 88% | 149 |
| K7VQQ1 | 4331400 | hydrolase, alpha/beta fold family protein | 88% | 459 |
| Q08277 | 4334919 | heat shock protein | 88% | 1195 |
| B4FME3 | 4339408 | expressed protein | 88% | 389 |
| B6SXY0 | 4332080 | DnaK family protein | 88% | 1023 |
| B6TPW4 | 4332744 | eukaryotic translation initiation factor 2 subunit beta | 88% | 457 |
| B8A0P3 | 4342077 | heat shock protein | 88% | 1240 |
| C0P4Q3 | 4334919 | heat shock protein | 88% | 1202 |
| B4FX40 | 4329339 | cysteine proteinase 1 precursor | 89% | 678 |
| B6SRE7 | 4347920 | peptidyl-prolyl cis-trans isomerase | 89% | 325 |
| B6UCG5 | 4326610 | psbP-related thylakoid lumenal protein 4, chloroplast precursor | 89% | 281 |
| B6TI78 | 4346090 | peptidyl-prolyl isomerase | 89% | 999 |
| Q43701 | 4325697 | hsp20/alpha crystallin family protein | 89% | 247 |
| B4FT54 | 4339609 | dnaJ domain containing protein | 90% | 579 |
| B6TQX0 | LOC_Os03g13450.2 | expressed protein | 91% | 308 |
| C0P6A4 | 4348209 | methyltransferase domain containing protein | 92% | 511 |
| K7UA57 | 4330730 | AAA family ATPase | 92% | 413 |
| B6UEB0 | 4337884 | LTPL104 - Protease inhibitor/seed storage/LTP family protein precursor | 93% | 152 |
| C0HG21 | 4345646 | ATP-dependent Clp protease adaptor protein ClpS containing protein | 93% | 213 |
| K7U7W9 | 4332690 | magnesium-chelatase | 93% | 2608 |
| P11143 | 4327388 | DnaK family protein | 93% | 1224 |
| B4FP70 | 4342486 | LSM domain containing protein | 93% | 243 |
| B6SP43 | 4350873 | ABC transporter, ATP-binding protein | 93% | 1068 |
| A4KA61 | 4348316 | profilin domain containing protein | 94% | 256 |
| B6SWZ1 | 4343590 | transporter family protein | 94% | 930 |
| K7TP06 | 4349044 | shikimate/quinate 5-dehydrogenase | 94% | 706 |
| B6SSK4 | OsI_17382 | 4F5 protein family protein | 94% | 94.7 |
| B4FL89 | 4328515 | chaperone protein clpB 1 | 95% | 702 |
| C0PDC7 | 4339343 | heat shock protein 101 | 96% | 1724 |
| C4J410 | 4327388 | DnaK family protein | 96% | 1280 |
| K7VJF3 | 4332420 | DnaK family protein | 96% | 1273 |
| Q6RYQ7 | 4339343 | heat shock protein 101 | 96% | 1712 |
| B4FIA6 | 4343519 | core histone H2A/H2B/H3/H4 | 99% | 233 |
| Q1KK93 | 3950734 | NADH dehydrogenase subunit 4 | 99% | 976 |
