# Supplementary material for: The Difference of Physiological and Proteomic Changes in Maize Leaves Adaptation to Drought, Heat, and Combined Both Stresses
Source: Front Plant Sci. 2016 Oct 26;7:1471. doi: 10.3389/fpls.2016.01471 (PMC5080359; doi:10.3389/fpls.2016.01471)
Supplement: Supplementary file 9 [file Table9.DOC]

**Table S9︱Maize proteins corresponding to rice proteins in network of protein interaction under combined stress.**

| Maize query sequence | Rice query sequence | STRING protein | Identity | Bitscore |
| --- | --- | --- | --- | --- |
| A4KA61 | 4348316 | profilin domain containing protein | 94% | 256 |
| P17571 | 4345795 | nitrate reductase | 88% | 1165 |
| B4F8F5 | 4330523 | fatty acid desaturase | 86% | 718 |
| B4FA43 | 4330554 | aspartic proteinase nepenthesin-1 precursor | 85% | 253 |
| B4FDE5 | 4335011 | CBS domain containing membrane protein | 76% | 176 |
| B4FFS8 | 4352868 | RNA recognition motif containing protein | 87% | 155 |
| B4FHM6 | 4337415 | protochlorophyllide reductase A, chloroplast precursor | 86% | 634 |
| B4FT63 | 4350255 | uncharacterized protein ycf53 | 68% | 358 |
| B4FTL9 | 4352201 | nodulin MtN3 family protein | 78% | 409 |
| B4FUV7 | 4328767 | expressed protein | 50% | 131 |
| B4FX40 | 4329339 | cysteine proteinase 1 precursor | 89% | 678 |
| P24067 | 4328075 | DnaK family protein | 96% | 1219 |
| Q9ATM5 | 4343119 | aquaporin protein | 91% | 533 |
| Q9FPK7 | 4331917 | inositol-3-phosphate synthase | 95% | 1019 |
| B4F8Z1 | 4332063 | pentatricopeptide repeat domain containing protein | 79% | 1024 |
| B4FAB3 | 4324152 | GTPase of unknown function domain containing protein | 86% | 488 |
| B4FI16 | 4325310 | phosphatidate cytidylyltransferase | 81% | 710 |
| B4FKD7 | LOC_Os09g26700.1 | choline/ethanolamine kinase | 83% | 604 |
| B4FKG5 | 4349876 | abscisic stress-ripening | 72% | 96.3 |
| B4FKX6 | 4347912 | oxidoreductase, short chain dehydrogenase/reductase family domain containing family | 82% | 540 |
| B4FL89 | 4328515 | chaperone protein clpB 1 | 95% | 702 |
| B4FME3 | 4339408 | expressed protein | 88% | 389 |
| B4FMW6 | 4331874 | aspartic proteinase nepenthesin precursor | 84% | 701 |
| B4FP20 | OsI_31806 | nuclear ribonuclease Z | 71% | 367 |
| B4FPQ2 | 4347851 | dihydroneopterin aldolase | 80% | 212 |
| B4FRG9 | 4334367 | fasciclin domain containing protein | 88% | 311 |
| B4FT54 | 4339609 | dnaJ domain containing protein | 89% | 462 |
| B4FUH2 | OsJ_03530 | aminotransferase, classes I and II, domain containing protein | 88% | 828 |
| B4G0P6 | 4344993 | expressed protein | 87% | 830 |
| B4G1K9 | 4347395 | photosystem I reaction center subunit, chloroplast precursor | 86% | 222 |
| B6SI29 | 4337607 | Core histone H2A/H2B/H3/H4 domain containing protein | 88% | 228 |
| B6SIA6 | 4342247 | expressed protein | 84% | 141 |
| B6SP43 | 4350873 | ABC transporter, ATP-binding protein | 93% | 1068 |
| B6SR73 | OsI_36451 | expressed protein | 81% | 200 |
| B6SRB1 | 4350556 | HVA22 | 80% | 231 |
| B6SSB7 | 4337600 | cysteine-rich repeat secretory protein 55 precursor | 76% | 355 |
| B6SSH9 | 4347709 | ribonuclease T2 family domain containing protein | 58% | 271 |
| B6ST41 | 4349616 | BTBN20 - Bric-a-Brac, Tramtrack, Broad Complex BTB domain with non-phototropic hypocotyl 3 NPH3 and coiled-coil domains | 63% | 597 |
| B6T026 | 4330227 | LTPL113 - Protease inhibitor/seed storage/LTP family protein precursor | 80% | 177 |
| B6TA17 | H0211A12.7 | kinesin heavy chain isolog | 69% | 355 |
| B6TB13 | 4349754 | anthocyanin 5-O-glucosyltransferase | 47% | 414 |
| B6TC25 | 4345825 | gibberellin receptor GID1L2 | 79% | 506 |
| B6TGK8 | 4345686 | omega-6 fatty acid desaturase, chloroplast precursor | 82% | 786 |
| B6TGW6 | 4328250 | activator of 90 kDa heat shock protein ATPase homolog | 76% | 290 |
| B6TI78 | 4346090 | peptidyl-prolyl isomerase | 89% | 999 |
| B6TJM1 | 4324442 | dehydrogenase | 95% | 715 |
| B6TK50 | 4334611 | armadillo/beta-catenin-like repeat containing protein | 88% | 663 |
| B6TLM5 | 4325643 | glutathione S-transferase | 71% | 319 |
| B6TQG2 | 4344249 | stress-related protein | 77% | 340 |
| B6TYT3 | OsI_13535 | cysteine protease 1 precursor | 73% | 459 |
| B6U1W0 | OsI_04983 | CBS domain-containing protein | 88% | 878 |
| B6UBQ9 | 4349089 | dehydration response related protein | 46% | 540 |
| B6UBW7 | 4336183 | uncharacterized glycosyltransferase | 79% | 1220 |
| B6UDP0 | 4327971 | OsPDIL1-4 protein disulfide isomerase PDIL1-4 | 79% | 826 |
| B6UFX4 | 4324498 | Core histone H2A/H2B/H3/H4 domain containing protei | 89% | 204 |
| B8A161 | 4339460 | NAP domain containing protein, putative, expressed | 84% | 553 |
| C0HE53 | OsI_32273 | uncharacterized protein PA4923, putative, expressed | 83% | 192 |
| C0HG57 | 4338768 | transketolase, putative, expressed; Catalyzes the acyloin condensation reaction between C atoms 2 and 3 of pyruvate and glyceraldehyde 3-phosphate to yield 1-deoxy-D-xylulose-5-phosphate (DXP) (By similarity). Essential for chloroplast development | 94% | 818 |
| C0P9L7 | 4341497 | copine-1, putative, expressed | 86% | 448 |
| C0PD01 | 4331484 | fasciclin-like arabinogalactan protein, putative, expressed | 55% | 359 |
| C0PNI2 | OsI_34221 | ATP synthase protein I related, putative, expressed | 89% | 566 |
| C4JAJ7 | 4347545 | Os9bglu31 - beta-glucosidase, dhurrinase, similar to G. max hydroxyisourate hydrolase | 83% | 866 |
| C4JBB8 | LOC_Os09g31486.1 | DnaK family protein | 93% | 1264 |
| D1MN58 | 4349876 | abscisic stress-ripening | 58% | 88.6 |
| K7U346 | 4126887 | ribulose bisphosphate carboxylase large chain precursor | 91% | 317 |
| K7USR3 | OsJ_01305 | magnesium-protoporphyrin IX monomethyl ester cyclase | 92% | 760 |
| K7UWZ6 | 4338790 | tubulin/FtsZ domain containing protein | 98% | 920 |
| K7UZF0 | 4327096 | peptide-N4-asparagine amidase A | 78% | 938 |
| K7VQU8 | 4331339 | DEAD-box ATP-dependent RNA helicase | 80% | 1045 |
| K7VZF7 | 4332853 | SNF2 family N-terminal domain containing protein | 79% | 2011 |
| O24595 | 4346877 | glutathione S-transferase | 63% | 283 |
| Q42446 | 4341942 | glycosyl hydrolases family 16 | 77% | 445 |
| Q5D1L6 | 4325560 | potassium channel KAT1 | 75% | 752 |
| P11143 | 4327388 | DnaK family protein | 93% | 1224 |
| Q08277 | 4334919 | heat shock protein | 88% | 1195 |
| Q42376 | 4326129 | late embryogenesis abundant protein, group 3 | 58% | 180 |
| A3KLI0 | 4350453 | dehydrin | 61% | 102 |
| A6YSM3 | OsJ_34075 | jacalin-like lectin domain containing protein | 43% | 130 |
| B4F7X5 | 4341121 | reticulon domain containing protein | 77% | 382 |
| B4F976 | 4332363 | hsp20/alpha crystallin family protein | 81% | 246 |
| B4F988 | 4340563 | OsFtsH6 FtsH protease, homologue of AtFtsH6 | 83% | 1066 |
| B4F9E8 | 4330933 | hsp20/alpha crystallin family protein | 74% | 233 |
| B4F9K4 | HSP18.0 | hsp20/alpha crystallin family protein | 78% | 220 |
| B4FGY0 | 4325302 | SGS domain containing protein | 77% | 346 |
| B4FIA6 | 4343519 | core histone H2A/H2B/H3/H4 | 99% | 233 |
| B4FLE3 | 4332979 | CS domain containing protein | 78% | 242 |
| B4FQS7 | 4330786 | heat shock 22 kDa protein, mitochondrial precursor | 76% | 305 |
| B4G1H1 | 4330265 | dehydrin | 57% | 190 |
| B4G250 | 4332357 | hsp20/alpha crystallin family protein | 87% | 253 |
| B5U8J8 | 4332506 | asparagine synthetase | 84% | 1055 |
| B6ETR5 | 4332506 | asparagine synthetase | 84% | 1054 |
| B6SID7 | 4326129 | late embryogenesis abundant protein, group 3 | 59% | 174 |
| B6SJR4 | 4341426 | OsFBK16 - F-box domain and kelch repeat containing protein | 73% | 536 |
| B6SP03 | OsJ_02789 | expressed protein | 81% | 62 |
| B6SQN7 | OsI_27179 | expressed protein | 74% | 340 |
| B6SXY0 | 4332080 | DnaK family protein | 88% | 1023 |
| B6SZ50 | OsI_19337 | retrotransposon protein, putative, LINE subclass | 61% | 238 |
| B6T2J9 | 4325697 | hsp20/alpha crystallin family protein | 81% | 232 |
| B6T3Q3 | OsJ_01972 | universal stress protein domain containing protein | 78% | 239 |
| B6T649 | 4330786 | heat shock 22 kDa protein, mitochondrial precursor | 76% | 298 |
| B6T8R8 | 4323890 | CPuORF25 - conserved peptide uORF-containing transcript | 87% | 922 |
| B6TA56 | 4340005 | histone H1 | 81% | 143 |
| B6TGE4 | 4329780 | protein binding protein | 58% | 306 |
| B6THJ5 | 4341866 | phosphosulfolactate synthase-related protein | 85% | 506 |
| B6TIJ3 | 4331115 | thioredoxin family protein | 79% | 405 |
| B6TIK3 | 4337833 | stress-related protein | 84% | 361 |
| B6TIP9 | HSP18.0 | hsp20/alpha crystallin family protein | 72% | 196 |
| B6TKI8 | 4342676 | expressed protein | 48% | 142 |
| B6TLK8 | 4332363 | hsp20/alpha crystallin family protein | 81% | 249 |
| B6TQD6 | 4340661 | hsp20/alpha crystallin family protein | 72% | 165 |
| B6TQX0 | LOC_Os03g13450.2 | expressed protein | 91% | 308 |
| B6TSV7 | 4345287 | late embryogenesis abundant group 1 | 71% | 187 |
| B6TTC8 | 4325697 | hsp20/alpha crystallin family protein | 87% | 249 |
| B6TWG6 | 4345944 | COP9 signalosome complex subunit 6a | 89% | 600 |
| B6TXS5 | 4351694 | photosystem I reaction center subunit N, chloroplast precursor | 76% | 206 |
| B6U100 | 4329388 | peptidyl-prolyl isomerase | 79% | 962 |
| B6UAU8 | 4349123 | cystathionine gamma-synthase | 89% | 774 |
| B6UET0 | 4333985 | peptidase, M50 family | 84% | 924 |
| B6UH30 | 4339065 | phosphatidylethanolamine-binding protein | 78% | 262 |
| B6UHH1 | 4349779 | expressed protein | 74% | 213 |
| B7ZEQ0 | 4332237 | hsp20/alpha crystallin family protein | 77% | 336 |
| B8A0P3 | 4342077 | heat shock protein | 88% | 1240 |
| C0HI30 | 4339812 | NAD dependent epimerase/dehydratase family protein | 89% | 653 |
| C0P4Q3 | 4334919 | heat shock protein | 88% | 1202 |
| C0P5X6 | 4351224 | AAA-type ATPase family protein | 80% | 690 |
| C0P732 | 4330134 | heat shock protein STI | 81% | 921 |
| C0PDC7 | 4339343 | heat shock protein 101 | 96% | 1724 |
| C4J0T9 | OsJ_04937 | haloacid dehalogenase-like hydrolase family protein | 83% | 411 |
| C4J410 | 4327388 | DnaK family protein | 96% | 1280 |
| C4J477 | 4330265 | dehydrin | 56% | 181 |
| E1U816 | 4341420 | endothelial differentiation-related factor 1 | 83% | 212 |
| F1DJV0 | 4327123 | transcription factor HY5 | 77% | 183 |
| K7TFB6 | 4352207 | GRAM domain containing protein | 85% | 422 |
| K7U4Y5 | 4329593 | decarboxylase | 93% | 736 |
| K7UFK0 | 4329593 | decarboxylase | 63% | 136 |
| K7UHS3 | OsI_21690 | retrotransposon protein | 35% | 236 |
| K7V5R0 | 4326516 | hexokinas | 86% | 608 |
| K7VBI0 | 4326743 | AAA-type ATPase family protein | 85% | 1382 |
| K7VJF3 | 4332420 | DnaK family protein | 96% | 1273 |
| K7VNX5 | 4327607 | cytochrome P450 | 83% | 836 |
| P93518 | LOC_Os01g47070.1 | glycosyl hydrolase | 72% | 429 |
| Q43701 | 4325697 | hsp20/alpha crystallin family protein | 89% | 247 |
| Q6RYQ7 | 4339343 | heat shock protein 101 | 96% | 1712 |
